## [Peer Review File · Nature Communications]

Reviewers' comments:

Reviewer #1 (Remarks to the Author):

Remote sensing can be used as a tool in biodiversity monitoring. Generally, the progress in this field is and will be important in conservation biology. This manuscript shows that radar (Sentinel-1) is equally good as the airborne laser scanning, which is what currently is often used. An advantage with using radar data is its complete geographic coverage and that it is freely available. Since I am not doing research in remote sensing I am not able to evaluate the novelty and quality of this manuscript from that perspective, but I comment the manuscript from the perspective of someone with a more general interest in conservation biology.

With remote sensing it is possible to monitor habitat loss and degradation (as well as improvement of habitat) and thus the potential for biodiversity in forests. However, throughout the manuscript, the authors should be clearer about the fact that such information should be combined with an understanding of ecological processes to predict species declines (or increases) at various spatial scales. I suggest several such changes below.

It is not clear what "biodiversity" the author want to monitor. In many cases, gamma diversity (total number of species within a region) is the most relevant. Maybe the approach suggested in this manuscript could be helpful when predicting gamma diversity, but nothing is mentioned about this. However, the main aim with the methodological development may be to predict biodiversity at some other scale. This should be better explained.

Overall, this study covers biodiversity in a broad sense, considering a large number of species groups that are relevant in biodiversity conservation in forests.

The data are not collected from any temperate forest in Europe, but all data are from biosphere reserves and national parks. This may affect the generality of the outcome, and the authors should therefore discuss that. There are European regions, where current forest biodiversity may be negatively affected by historically poor conditions in terms of the amount of forest or air pollution. It may also be that forest habitats are so fragmented that species are absent even though the substrates are present at a local scale, due to an impoverished regional species pool. It may also be that exotic tree species, with a low number of species associated with them, are far more abundant in other regions than those studied. All these factors may make predictions of biodiversity from radar data more difficult elsewhere than in the studied types of forest landscapes.

Generally, for arthropods the sample size (one pitfall trap and one flight interception trap) is very small. It means that the most abundant species are collected, and also that there is some chance of finding other species, but it is still only a rather small sample in comparison to the total number of species present at each site. However, I could not find any reason why we should expect this to generate any biases in the comparison of the remote sensing methods.

The focus on necrophagous beetles was unexpected, and should be commented by the authors.

Are they of conservation concern, and are the methods used really suitable for sampling this group of species?

For lichens, completely different substrates have been surveyed in different parts of the study (see Supplementary material, LL417-426). Bark, rocks deadwood and soil were surveyed in some plots, while in other "single trees" or "stems" (what is the difference between these two categories?) have been surveyed. Many species are specialised to certain substrates, and thus this sampling strategy will most likely generate clear differences in species richness and species composition just due to the differences in sampling between plots.

Methods. The mean temperature and precipitation are not the most important factors that have to be presented for each region. Could be deleted.

LL. 409-413. Something more is needed in the answer to this question: "how well can changes in biodiversity be monitored?" (only by using habitat quality variables alone). One important aspect is that extinction debts may occur, and then either habitat quality can decrease without a decrease in biodiversity (when an extinction debt is formed) or biodiversity can decrease without a decrease in habitat quality (when an extinction debt is paid). Furthermore, climate change may also change the relationship between biodiversity and habitat quality.

Some detailed comments:

- LL. 61. It provides indicators on habitat loss, rather than indicators on species declines.
- LL. 81-83. It is important to be clear about that remote sensing do not measure biodiversity.
- LL. 382-383. Actually not representative for all temperate forest ecosystems of Central Europe.
- LL. 387-404 This gives probably a very incomplete picture of this subject, and could thus be shortened.

Reviewer #2 (Remarks to the Author):

This manuscript makes an impressive contribution to the field of remote sensing of biodiversity. The work based on Sentinel-1 radar data is novel, the datasets comprehensive, the analyses state-of-the-art, and the manuscript well written. The authors made my job easy! I am enthusiastic about the manuscript, and learn a lot reading it.

General comments:

- the description of the way the radar data were analyzed is superficial. I realize that length limitations in the main manuscript preclude detailed descriptions there, but sure had hoped that the lengthy SI would provide more than just a table of the variables that were calculated. I strongly suggest to add a detailed description how the radar data were processed, including any code, so that others can follow, and potentially replicate the approach
- when comparing the radar and lidar data, I suggest comparing all individual variables directly in pairwise comparisons in addition to the CCoA

Minor comments:

- the abstract is rather vague and does not include any numerical findings
- I suggest using the term lidar instead of of ALS throughout the manuscript

Response to Reviewers

Manuscript ID NCOMMS-19-13443

entitled "Radar vision in the mapping of forest biodiversity from space "

02 August 2019

Summary of main changes

1. Additional robustness test of our arthropod results against the sampling completeness

It has been questioned if one window and one pitfall trap are sufficient to represent the local community. The critical point here is not the number of traps but the number of individuals collected locally. In order to test the robustness of our arthropod results, we re-ran the richness and community composition analyses using a subset of plots with sample coverage more than 90%, 80% and 70%. These additional analyses support the robustness of our original findings and are shown now in the supplement (Figure S22).

2. Details about the radar data processing

We added more details about radar data processing in Supplementary Method S3. It includes S3.1 Download of the GRDH product from the ESA Sentinel Data Hub (DHuS), S3.2 Process Sentinel 1 GRDH data to gamma_0, and S3.3 Derivation of pixel- and neighborhood-based summary statistics. We uploaded corresponding batch processing configuration file for the SNAP toolbox software and the R script for pixel- and summary statistics at GitHub. We will publicise it after revisions.

Reviewer #1

Remote sensing can be used as a tool in biodiversity monitoring. Generally, the progress in this field is and will be important in conservation biology. This manuscript shows that radar (Sentinel-1) is equally good as the airborne laser scanning, which is what currently is often used. An advantage with using radar data is its complete geographic coverage and that it is freely available.

Since I am not doing research in remote sensing I am not able to evaluate the novelty and quality of this manuscript from that perspective, but I comment the manuscript from the perspective of someone with a more general interest in conservation biology.

With remote sensing it is possible to monitor habitat loss and degradation (as well as improvement of habitat) and thus the potential for biodiversity in forests. However, throughout the manuscript, the authors should be clearer about the fact that such information should be combined with an understanding of ecological processes to predict species declines (or increases) at various spatial scales. I suggest several such changes below.

It is not clear what "biodiversity" the author want to monitor. In many cases, gamma diversity (total number of species within a region) is the most relevant. Maybe the approach suggested in this manuscript could be helpful when predicting gamma diversity, but nothing is mentioned about this.

However, the main aim with the methodological development may be to predict biodiversity at some other scale. This should be better explained.

→ *We thank for this comment which helped to clarify in our manuscript how the results can be used to estimate alpha, beta and gamma diversity. The main interest of this work was related to local diversity, in particular 1-ha scale, but also the diversity among patches. The first was addressed by taxonomic richness and phylogenetic diversity, while the beta diversity was addressed by the community approach. Both together form the gamma diversity. We clarified the concepts of alpha, beta and gamma diversity and its relation beforehand and explained what we investigated among the various biodiversity aspects in the Introduction and Methods. In addition, we extended our Discussion how the new ability of predicting alpha and beta diversity over large areas by Sentinel-1 opens the door for large scale gamma diversity estimations.*

(Introduction: lines 83-86)

The unsolved challenge here is to monitor the diversity within (α -diversity) and between habitats (β -diversity) and even challenging one is to monitor area-wide biodiversity across large areas and get an estimate of total (γ -) diversity of landscapes.

(Introduction: lines 134-142)

Our study began with a comparison of the ecological application of radar (henceforth, “Sentinel-1” is referred to as “radar”) metrics vs. the well-established ALS metrics in providing a better understanding of habitat structure in forest ecosystems. A suite of ground-truth taxonomic and phylogenetic biodiversity measures covering within forest stand (α -) and among forest stand (β -) diversity from a broad range of trophic levels and taxa (henceforth “functional groups”) was then modelled using either ALS data or time-series radar data to explore the extent to which rich time-series radar data can be used to represent ecologically meaningful gradients of habitat conditions in temperate forests.

(Methods: lines 543-549)

Among the various aspects of biodiversity, alpha diversity measures the diversity of species within each plot and beta diversity the difference of species composition among these plots. Gamma diversity is a measure of the overall diversity within a region, a product of the alpha diversity for all the plots within a region and the beta diversity among them, thus often called as regional diversity. At our 1-ha local scale, species richness and phylogenetic diversity, as alpha diversity, and species composition, base of calculating beta diversity, were investigated.

(Discussion: lines 378-381)

Furthermore, as gamma diversity could be estimated as a product of alpha and beta diversity, Sentinel-1 can be applied to estimate gamma diversity even for large landscapes where ground estimations will stay impossible.

Overall, this study covers biodiversity in a broad sense, considering a large number of species groups that are relevant in biodiversity conservation in forests.

The data are not collected from any temperate forest in Europe, but all data are from biosphere reserves and national parks. This may affect the generality of the outcome, and the authors should therefore discuss that. There are European regions, where current forest biodiversity may be negatively affected by historically poor conditions in terms of the amount of forest or air pollution. It may also be that forest habitats are so fragmented that species are absent even though the substrates are present at a local scale, due to an impoverished regional species pool. It may also be that exotic tree species, with a low number of species associated with them, are far more abundant in other regions than those studied. All these factors may make predictions of biodiversity from radar data more difficult elsewhere than in the studied types of forest landscapes.

→ *We thank the reviewer for this comment which showed that our description of the regions was misleading. All studied regions comprised a large gradient of management intensity on the stand scale ranging from natural old-growth forests to intensively managed forest stands, including plantations of tree species non-natural to the sites. In the Biodiversity Exploratories, plots were selected to represent a gradient from unmanaged (mature forest not used for 20-70 years), uneven-aged selection forests (trees harvested selectively), to age-class forests (harvested at 80–120 year intervals) of either natural broadleaf or non-natural conifer plantations to explore the effects of land use on biodiversity¹. The BioKlim-Project located in the Bavarian Forest NP also stratified their plots along the gradient of forest management intensity (from the extremes of continuous logging activity to strictly protected forests within in the national park area)². Here it is important to know that the whole area of the national park was an intensively used common spruce dominated production forests when the park was established. In half of the park, forest management was still going on when data were sampled. Lastly, the Steigerwald forest is neither a biosphere reserve nor a national park, but exhibits the steepest gradient of land-use intensity within a beech forest in Europe over the last 70 years³. Therefore, in terms of intensity of human impact, our plots are not biased to the unmanaged forest. However, we agree with the reviewer, that we should be careful to generalize our outcome to other temperate forest outside of either our regional species pool or geographic neighbouring area. That is why we discussed the needs for geographically stratified national biodiversity monitoring system in the last section of the Discussion. Nevertheless, the external validation of the radar models using independent external data collected from areas outside the five training areas demonstrated the promising predictive ability of radar-derived data. Among the external validation plots, three regions, i.e., Bavarian Forest, Berchtesgaden, and Brandenburg, are located outside of biosphere reserves and national parks. To make these points clearer, we modified the Methods section as follows.*

(Methods: lines 454-469)

*The three regions of the Biodiversity Exploratories cover different forest management intensities: unmanaged old-growth forests, managed uneven-aged forests and managed age-class forests including natural broad-leaved tree species, mainly European beech *Fagus sylvatica*, and non-natural conifer*

plantations, i.e. Norway spruce Picea abies and Scots pine Pinus sylvestris. The Steigerwald region (STE) is located in a hilly area (400–520 m a.s.l.) in central Germany (N 49° 80'–49° 94'; E 10° 45'–10° 62') with a large gradient of broadleaf forest use intensity. It is dominated by F. sylvatica. The Bavarian Forest National Park region (BAY) is located in a mountainous area (710–1530 m a.s.l.) (N 48° 91'–49° 20'; E 13° 19'–13° 45'). The dominant tree species are P. abies and F. sylvatica. Half of the area, at the time of data sampling, was dominated by common production forests, while the other half was covered by strictly protected area with intensive natural disturbances or old-growth stands. Thus, the 463 plots included a long gradient of forest management intensity on the stand scale ranging from unmanaged old-growth forests to intensively managed forests.

Generally, for arthropods the sample size (one pitfall trap and one flight interception trap) is very small. It means that the most abundant species are collected, and also that there is some chance of finding other species, but it is still only a rather small sample in comparison to the total number of species present at each site. However, I could not find any reason why we should expect this to generate any biases in the comparison of the remote sensing methods.

→ Sampling completeness is indeed an important consideration in arthropod sampling. To represent the local community, it is important to collect sufficient individuals, which is more important than the number of traps installed. In previous studies we have shown that such an approach of one pitfall and one flight trap for the whole vegetation period is suitable if enough individuals were collected⁴. Moreover, in another study it has been shown that this kind of community sampling represent well not the very small scale but the stand scale with a radius of 30-50 meters around the trap⁵, which fit very well to our one hectare plot. However, previous studies have shown an increasing R^2 of richness and community composition along with an increase of the number of sampled individuals⁴. Therefore, to test the robustness of our results against the sampling completeness, we conducted additional analyses. We re-ran the richness and community composition analyses using a subset of plots with sample completeness reaching 90%, 80% and 70% according to the method developed by Chao and Jost⁶. This takes into account the variation in true diversity of local communities. They support the results of our original analyses (see Figure S22). We added the explanation in the Results, Methods, and Supplementary.

(Results: lines 205-208)

To check the robustness of our results of arthropods for sample size we reanalysed the data on a subset of plots with sufficient sample completeness. These findings corroborated the findings of the total data set (Supplement Fig. S22).

(Methods: lines 619-626)

Previous studies have shown that the R^2 of biodiversity measures increase with sampling size in arthropod samples collected by flight interception and pitfall traps up to a sample size of sufficient individuals⁴.

*Chao and Jost*⁶ introduced the sample completeness to standardize the comparability of diversity among communities. Therefore to check the robustness of our results for arthropods against the sampling completeness we re-ran the richness and community composition analyses using a subset of plots with sample coverage more than 90%, 80% and 70%.

(Figure S22)

Figure S 12 Robustness test of assemblage habitat models (boosted generalised additive models) using the ALS (orange bars) and radar (blue bars) data sets against different subsets of plots in comparison to total data. The shaded bars represent R^2 (coefficient of determination) derived from the models with a subset of plots over (a) sample coverage 90%, (b) sample coverage 80%, (c) sample coverage 70% using the method by Chao and Jost³ to calculate sample completeness for each sample based on the individuals with function *iNext* in the add-on package *iNext* in R. As the number of plots over sample coverage 90% of saproxyllic beetle is too low for cross validation, we excluded this analysis. Note that all analyses matched very well the finding of our total data, underlying the sufficient data of our original analyses (Fig. 3).

The focus on necrophagous beetles was unexpected, and should be commented by the authors. Are they of conservation concern, and are the methods used really suitable for sampling this group of species?

→ **There is growing evidence that biodiversity plays an important role in ecosystem functions and services. In our multitaxon approach, we aimed therefore to cover as many different taxonomical – functional groups as possible⁷. Within the trophic level of decomposers we were interested to have not only the wood-inhabiting fungi but also an insect group driving the decomposition of heterotrophic necromass. We have long experience that our trap system**

collects necrophagous beetles (partly attracted by insects in the trap itself, because traps were emptied only once per month) in high numbers of species and individuals. Hence, we included the necrophagous beetles in the analysis as one functional important group in ecosystems, and the strong correlations with the structure confirmed our choice.

For lichens, completely different substrates have been surveyed in different parts of the study (see Supplementary material, LL417-426). Bark, rocks deadwood and soil were surveyed in some plots, while in other "single trees" or "stems" (what is the difference between these two categories?) have been surveyed. Many species are specialised to certain substrates, and thus this sampling strategy will most likely generate clear differences in species richness and species composition just due to the differences in sampling between plots.

→ Thanks for this comment. Here the description how species were sampled and which species were included in our analyses was incorrect. From the Biodiversity Exploratories, we actually did only include species that were recorded on woody objects, that is, corticolous and lignicolous species. You are absolutely right, including lichens growing on soil and stone, which were not recorded in Steigerwald and Bavarian Forest, would have had a huge effect. We greatly apologize, that we forgot to include this important note within the Supplement. We corrected this now and also specified the wording in Supplementary S 1.2.5.

(Supplementary S 1.2.5)

In the Exploratories plots, lichens on four different substrates (bark [noting the tree species], rocks, deadwood and soil) within a 20 m × 20 m quadrats were recorded during a single session between 2007 and 2008⁸. Lichens were recorded in one round on all trees and dead wood stems and logs (hereafter referred to as stems) within a 14 m × 14 m plot in 2017 in the Steigerwald forest and within an 8-m radius from August to November 2007 in the Bavarian Forest National Park, respectively. In the Bavarian Forest National Park, 1–10 stems (average: 5 stems per plot) were examined, depending on stem availability (for details, see Moning, et al. ⁹). Because lichens which were observed on rocks and soil were only recorded on the Exploratories plots, they were excluded from our analysis.

Methods. The mean temperature and precipitation are not the most important factors that have to be presented for each region. Could be deleted.

→ We deleted the phrases.

LL. 409-413. Something more is needed in the answer to this question: "how well can changes in biodiversity be monitored?" (only by using habitat quality variables alone). One important aspect is that extinction debts may occur, and then either habitat quality can decrease without a decrease in biodiversity (when an extinction debt is formed) or biodiversity can decrease without a decrease in

habitat quality (when an extinction debt is paid). Furthermore, climate change may also change the relationship between biodiversity and habitat quality.

→ ***We agree that it is important to add the issue of possible extinction debts in our discussion when we discuss the needs to be investigated for biodiversity-change monitoring. We modified the sentence in the Discussion.***

(Discussion: lines 423-426)

Furthermore, thresholds for the detection of alterations in habitat conditions that trigger positive and negative biodiversity outcomes, the time delay in extinction after the habitat degradation and synergistic process with other threats such as climate change must still be defined.

Some detailed comments:

LL. 61. It provides indicators on habitat loss, rather than indicators on species declines.

→ ***We clarified the sentence in the Abstract as follows.***

(Abstract: lines 61-63)

Recent progress in remote sensing provides much-needed, large-scale spatio-temporal information on habitat structures important for biodiversity conservation.

LL. 81-83. It is important to be clear about that remote sensing do not measure biodiversity.

→ ***We clarified the sentence in the Introduction as follows.***

(Introduction: lines 86-90)

However, over the last decade, advances in remote sensing have led to an exponential increase in the use of these technologies, including in ecological investigations, and a recognition of their potential in obtaining reliable and frequent updates on the spatial information required to monitor biodiversity over larger areas, information that is essential for conservationists.

LL. 382-383. Actually not representative for all temperate forest ecosystems of Central Europe.

→ ***We consider our data to represent the major parts of Central European forests excluding azonal forests as floodplains from low lands to mountains and from natural stands to intensively managed ones.***

(Discussion: lines 393-394)

Although our study covered five forested regions, these were representative only of the major temperate forest ecosystems of Central Europe.

LL. 387-404 This gives probably a very incomplete picture of this subject, and could thus be shortened.

→ ***We have shortened it in the Discussion (lines 403-410) as suggested.***

Reviewer #2

This manuscript makes an impressive contribution to the field of remote sensing of biodiversity. The work based on Sentinel-1 radar data is novel, the datasets comprehensive, the analyses state-of-the-art, and the manuscript well written. The authors made my job easy! I am enthusiastic about the manuscript, and learn a lot reading it.

General comments:

- the description of the way the radar data were analyzed is superficial. I realize that length limitations in the main manuscript preclude detailed descriptions there, but sure had hoped that the lengthy SI would provide more than just a table of the variables that were calculated. I strongly suggest to add a detailed description how the radar data were processed, including any code, so that others can follow, and potentially replicate the approach

→ ***We added more details about radar data processing in Supplementary Method S3. It includes S3.1 Download of the GRDH product from the ESA Sentinel Data Hub (DHuS), S3.2 Process Sentinel 1 GRDH data to gamma_0, and S3.3 Derivation of pixel- and neighborhood-based summary statistics. We uploaded corresponding batch processing configuration file for the SNAP toolbox software and the R script for pixel- and summary statistics at GitHub (<https://github.com/So-YeonBae/Sentinel1-Biodiversity>). We will publicise it after revisions.***

- when comparing the radar and lidar data, I suggest comparing all individual variables directly in pairwise comparisons in addition to the CCoA

→ ***We added Figure S 3 Correlation matrix between the radar metrics and the ALS metrics in Supplementary figures.***

Figure S 2 Correlation matrix between the radar metrics and the ALS metrics at the significance level $p < 0.05$.

Minor comments:

- the abstract is rather vague and does not include any numerical findings

→ **We included numeric findings in the Abstract now.**

(Abstract: lines 67-70)

Modelling different facets of biodiversity reveals that radar performs as well as ALS; median R^2 over twelve taxa by ALS and radar are 0.51 and 0.57 respectively for a measure representing assemblage composition.

- I suggest using the term lidar instead of of ALS throughout the manuscript

→ **Thank you for your comments. However, we would like to stick to use the term ALS, because lidar can be used for both terrestrial lidar, airborne lidar, and spaceborne lidar.**

References

- 1 Fischer, M. *et al.* Implementing large-scale and long-term functional biodiversity research: The Biodiversity Exploratories. *Basic and Applied Ecology* **11**, 473-485, doi:<https://doi.org/10.1016/j.baae.2010.07.009> (2010).
- 2 Bässler, C., Förster, B., Moning, C. & Müller, J. The BIOKLIM project: biodiversity research between climate change and wilding in a temperate montane forest : the conceptual framework. *Waldökologie, Landschaftsforschung und Naturschutz*, 21-34 (2009).
- 3 Zytynska, S. E. *et al.* Minimal effects on genetic structuring of a fungus-dwelling saproxylic beetle after recolonisation of a restored forest. *Journal of Applied Ecology* **55**, 2933-2943, doi:10.1111/1365-2664.13160 (2018).
- 4 Müller, J. & Brandl, R. Assessing biodiversity by remote sensing in mountainous terrain: the potential of LiDAR to predict forest beetle assemblages. *Journal of Applied Ecology* **46**, 897-905, doi:10.1111/j.1365-2664.2009.01677.x (2009).
- 5 Leutner, B. *Space for Communities: Quantifying Data Requirements for Remote Sensing Based Habitat Modeling*, University Würzburg, (2018).
- 6 Chao, A. & Jost, L. Coverage-based rarefaction and extrapolation: standardizing samples by completeness rather than size. *Ecology* **93**, 2533-2547, doi:10.1890/11-1952.1 (2012).
- 7 Seibold, S., Cadotte, M. W., Maclvor, J. S., Thorn, S. & Müller, J. The Necessity of Multitrophic Approaches in Community Ecology. *Trends in Ecology & Evolution* **33**, 754-764, doi:<https://doi.org/10.1016/j.tree.2018.07.001> (2018).
- 8 Boch, S., Prati, D., Hessenmöller, D., Schulze, E.-D. & Fischer, M. Richness of Lichen Species, Especially of Threatened Ones, Is Promoted by Management Methods Furthering Stand Continuity. *PLOS ONE* **8**, e55461, doi:10.1371/journal.pone.0055461 (2013).
- 9 Moning, C. *et al.* Lichen diversity in temperate montane forests is influenced by forest structure more than climate. *Forest Ecology and Management* **258**, 745-751, doi:<https://doi.org/10.1016/j.foreco.2009.05.015> (2009).

REVIEWERS' COMMENTS:

Reviewer #1 (Remarks to the Author):

I am happy with all responses to my previous comments and the changes which were done in the manuscript.

Reviewer #2 (Remarks to the Author):

All my concerns have been addressed to my satisfaction. Congratulations on a very nice manuscript!

Point-by-point Response

Manuscript ID NCOMMS-19-13443A

entitled "Radar vision in the mapping of forest biodiversity from space "

05 September 2019

Summary of main changes

- I. We provide a separate Source Data file named "Source Data.xlsx", complied excel sheets of source data for Figures 1, 4 and Supplementary Figures 2, 5, 18, 19.***
- II. With respect to display items, we followed the editor's recommendation adding brief titles and colour scale explanation in legends, applying colour blindness palettes, and indicating our license to use the clip-arts in figures in point-to-point response.***
- III. The batch processing configuration file for the SNAP toolbox software and the R script for pixel- and summary statistics are provided at Supplementary Software as a ZIP file "Sentinel1_ExampleData2.zip" as well.***
- IV. We added a Supplementary Table 11 for the details of permits received for fieldwork.***

REVIEWERS' COMMENTS

Reviewer #1 (Remarks to the Author):

I am happy with all responses to my previous comments and the changes which were done in the manuscript.

Reviewer #2 (Remarks to the Author):

All my concerns have been addressed to my satisfaction. Congratulations on a very nice manuscript!

→ ***We appreciate your time and effort in considering our work.***