# Supplementary material for: Radar vision in the mapping of forest biodiversity from space
Source: Nat Commun. 2019 Oct 18;10:4757. doi: 10.1038/s41467-019-12737-x (PMC6802221; doi:10.1038/s41467-019-12737-x)
Supplement: Supplementary file 1 — Supplementary Information [file 41467_2019_12737_MOESM1_ESM.pdf]

Radar vision in the mapping of forest biodiversity from space

Bae et al.

Supplementary Information: list of material provided

Supplementary Figures

Supplementary Figure 1 Distribution map of the plots.

Supplementary Figure 2 Principal components analysis of the metrics derived from the airborne laser scanning (ALS) and radar data sets.

Supplementary Figure 3 Correlation matrix between the radar metrics and the ALS metrics at the significance level $p<0.05$.

Supplementary Figure 4 Visual comparison of the winter VH and the Corine Land Cover Map of the Schorfheide-Chorin area.

Supplementary Figure 5 Non-metric multidimensional scaling (NMDS) of 12 functional groups.

Supplementary Figure 6 Importance of the variables in the assemblage habitat models (boosted generalised additive models, GAMs) of vascular plants according to the ALS (first row) and radar (second row) data sets.

Supplementary Figure 7 Importance of the variables in the assemblage habitat models (boosted GAMs) of bryophytes according to the ALS (first row) and radar (second row) data sets.

Supplementary Figure 8 Importance of the variables in the assemblage habitat models (boosted GAMs) of lichens according to the ALS (first row) and radar (second row) data sets.

Supplementary Figure 9 Importance of the variables in the assemblage habitat models (boosted GAMs) of phytophagous beetles according to the ALS (first row) and radar (second row) data sets.

Supplementary Figure 10 Importance of the variables in the assemblage habitat models (boosted GAMs) of moths according to the ALS (first row) and radar (second row) data sets.

Supplementary Figure 11 Importance of the variables in the assemblage habitat models (boosted GAMs) of saproxylic beetles according to the ALS (first row) and radar (second row) data sets.

Supplementary Figure 12 Importance of the variables in the assemblage habitat models (boosted GAMs) of fungi according to the ALS (first row) and radar (second row) data sets.

Supplementary Figure 13 Importance of the variables in the assemblage habitat models (boosted GAMs) of necrophagous beetles according to the ALS (first row) and radar (second row) data sets.

Supplementary Figure 14 Importance of the variables in the assemblage habitat models (boosted GAMs) of spiders according to the ALS (first row) and radar (second row) data sets.

Supplementary Figure 15 Importance of the variables in the assemblage habitat models (boosted GAMs) of carabid beetles according to the ALS (first row) and radar (second row) data sets.

Supplementary Figure 16 Importance of the variables in the assemblage habitat models (boosted GAMs) of birds according to the ALS (first row) and radar (second row) data sets.

Supplementary Figure 17 Importance of the variables in the assemblage habitat models (boosted GAMs) of bats according to the ALS (first row) and radar (second row) data sets.

Supplementary Figure 18 The assemblage composition of (a) birds and (b) saproxylic beetles in the different sampling regions as evaluated by NMDS.

Supplementary Figure 19 Scatter plots of the observed vs. the predicted values from the training and validation data of (a) birds and (b) saproxylic beetles.

Supplementary Figure 20 Predicted maps of the Bavarian Forest National Park.

Supplementary Figure 21 Importance of the variables in the coniferous ratio models (boosted GAMs) according to the ALS (first row) and radar (second row) data sets.

Supplementary Figure 22 Robustness test of assemblage habitat models (boosted generalised additive models) using the ALS (orange bars) and radar (blue bars) data sets against different subsets of plots in comparison to total data.

Supplementary Tables

Supplementary Table 1 Radar metrics

Supplementary Table 2 ALS data sources

Supplementary Table 3 ALS metrics

Supplementary Table 4 Summary of the results of the canonical correlation analysis.

Supplementary Table 5 Pearson's correlation coefficient for the ALS metrics vs. axes 1–9 of the canonical correlation analysis (Ycan1–9).

Supplementary Table 6 Pearson's correlation coefficient for the radar metrics vs. axes 1–9 of the canonical correlation analysis (Xcan 1–9).

Supplementary Table 7 Cross-validated performance (R^2, coefficient of determination) of the assemblage habitat models (boosted generalised additive models, GAMs) in the fixed effects models using the ALS and radar data sets.

Supplementary Table 8 Cross-validated performance (root mean square error) of the assemblage habitat models (boosted GAMs) in the fixed effects models using the ALS and radar data sets.

Supplementary Table 9 Cross-validated performance (R^2) of the assemblage habitat models (boosted GAMs) in the mixed effects models using the ALS and radar data sets.

Supplementary Table 10 Loss of R^2 in the assemblage habitat models by region.

Supplementary Table 11 Permits received for fieldwork

Supplementary Table 12 References for the basic topologies of the phylogenetic trees of 12 functional groups

Supplementary Table 13 Example for running the pre-processing from a batch command line.

Supplementary Notes

Supplementary Note 1 Sources of species data and detailed descriptions of the sampling methods

Supplementary Note 2 Description of the calculations of phylogenetic diversity

Supplementary Note 3 Sentinel-1 data pre-processing and pixel-based summary statistics

Supplementary Figures

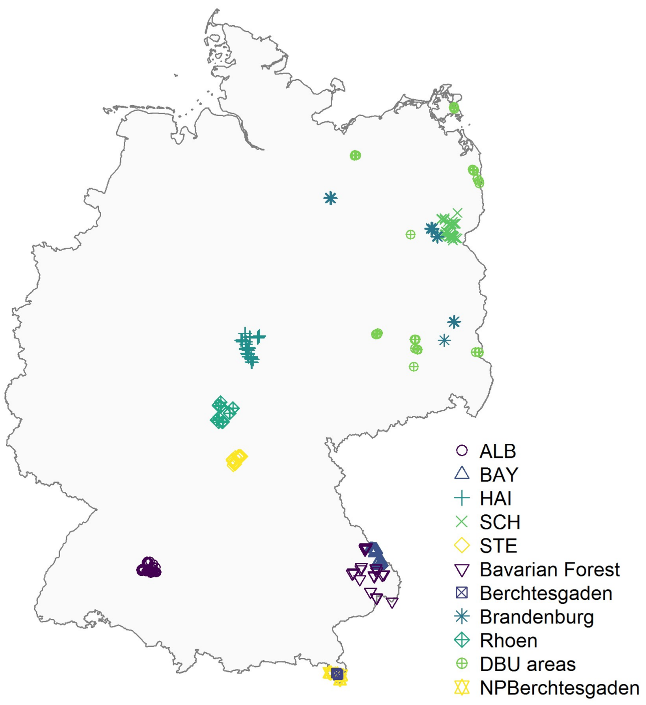

Supplementary Figure 1 Distribution map of the plots. The first five regions are training regions; the remainder are the regions located outside the training regions and used in external validation.

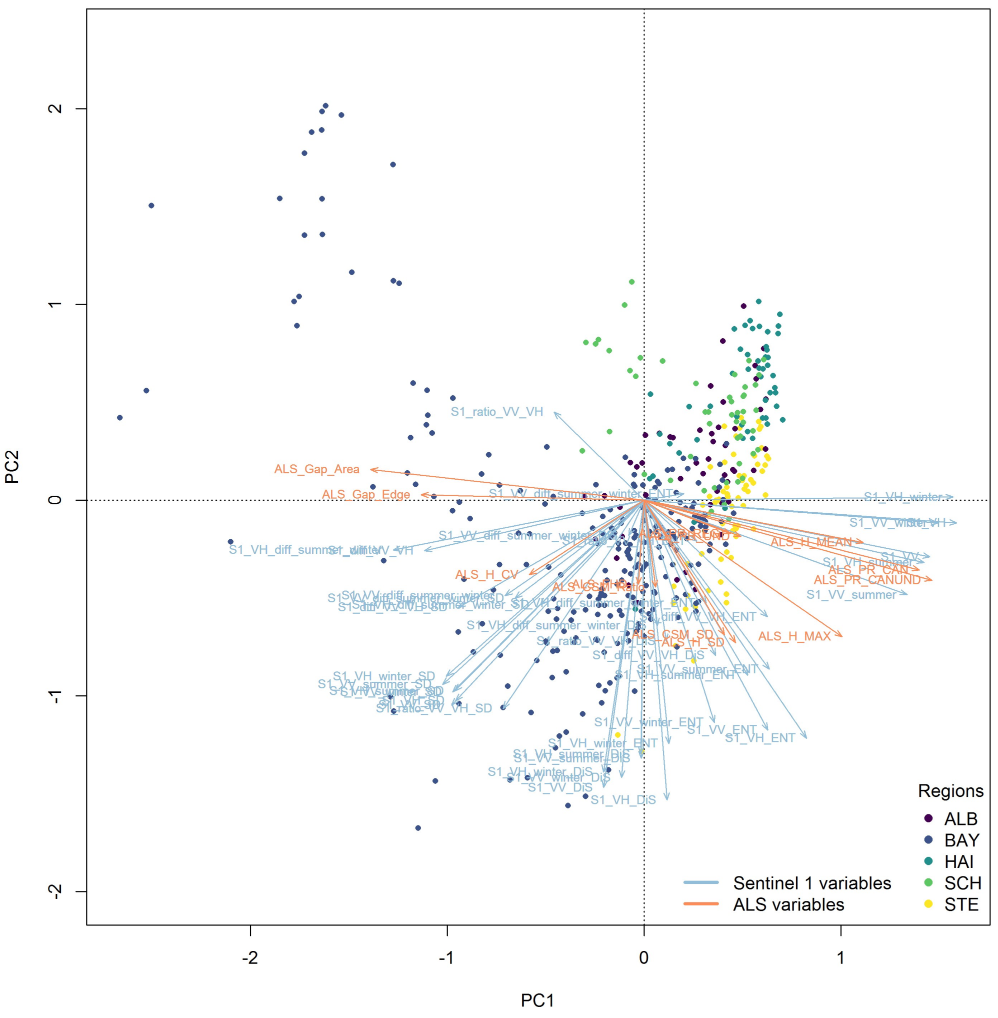

Supplementary Figure 2 Principal components analysis of the metrics derived from the airborne laser scanning (ALS) and radar data sets. The first axis explained 24.40% of the variation in the 53 variables, 40 from radar and 13 from ALS, and was associated with forest maturity, e.g. the penetration ratio of canopy-understorey layers ($PR_{>2m}$) and the total area of forest gaps (Gap_{Area}), determined from the ALS data and the winter median of the vertically-sent, horizontally-received radar pulses (VH_{winter}) and of the vertically-sent, vertically-received radar pulses (VV_{winter}). The second axis explained 20.92% of the variation and was associated with structural heterogeneity: e.g. the standard deviation of the vegetation height (H_{SD}) and of the canopy surface height (CSH_{SD}) from the ALS data and the dissimilarity of VH and VV with respect to neighbouring pixels ($VH_{Diss}$and VV_{Diss}). Among the radar metrics, the standard deviation and image texture (i.e., dissimilarity and entropy) of the backscatters were closely related to the structural heterogeneity axes. The standard deviation of the vegetation returns (H_{SD}) and of the canopy surface height (CSH_{SD}) as well as the foliage height diversity (FHD) in the ALS metrics and the image texture of the backscatters in the radar metrics composed the second principle component. Source data are provided as a Source Data file.

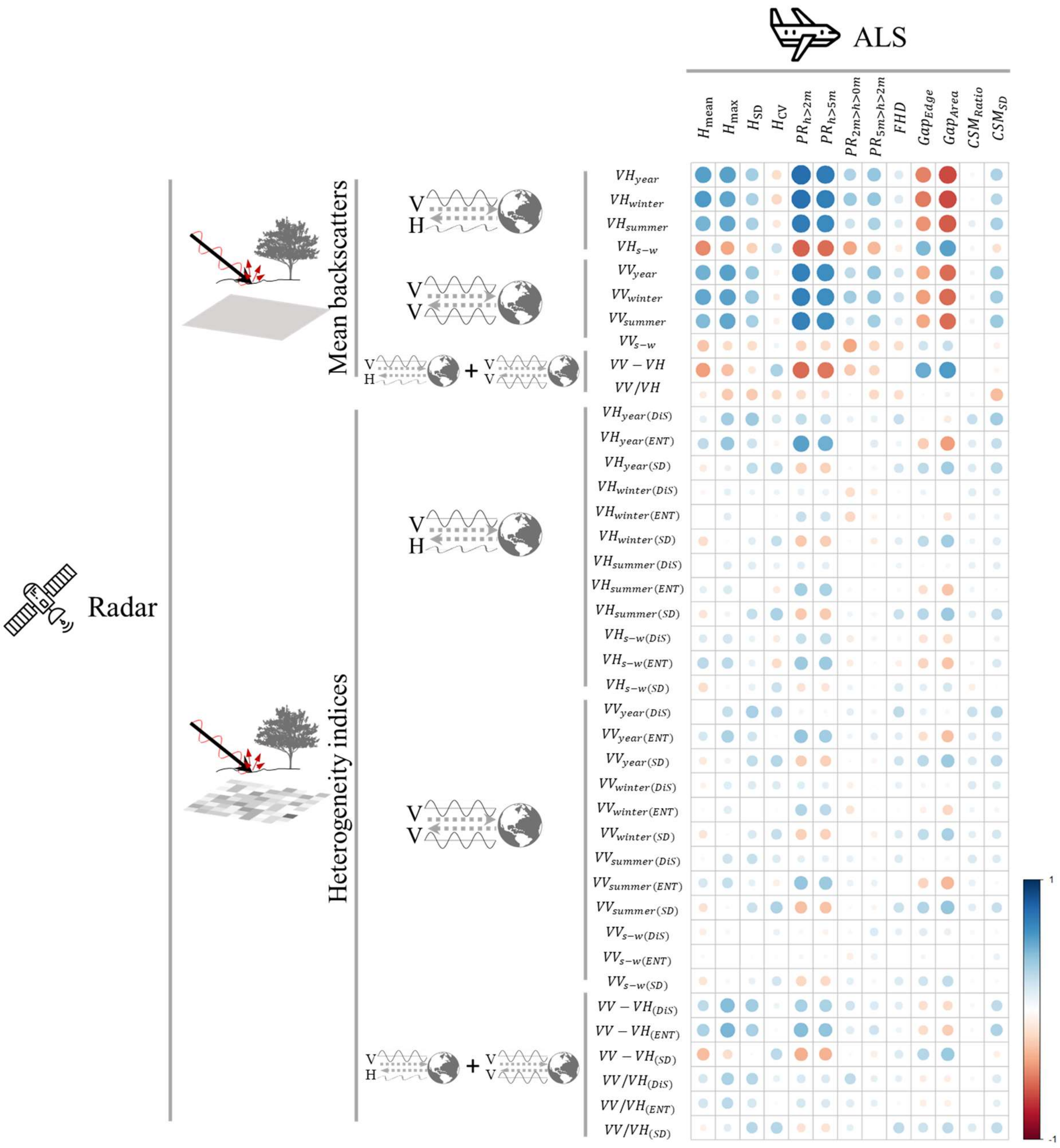

Supplementary Figure 3 Correlation matrix between the radar metrics and the ALS metrics at the significance level p<0.05. Positive correlations are displayed in blue and negative correlations in red color. Color intensity and the size of the circle are proportional to the correlation coefficients.

Winter VH of the Schorfheide-Chorin area	Corine Land Cover Map of the Schorfheide-Chorin area

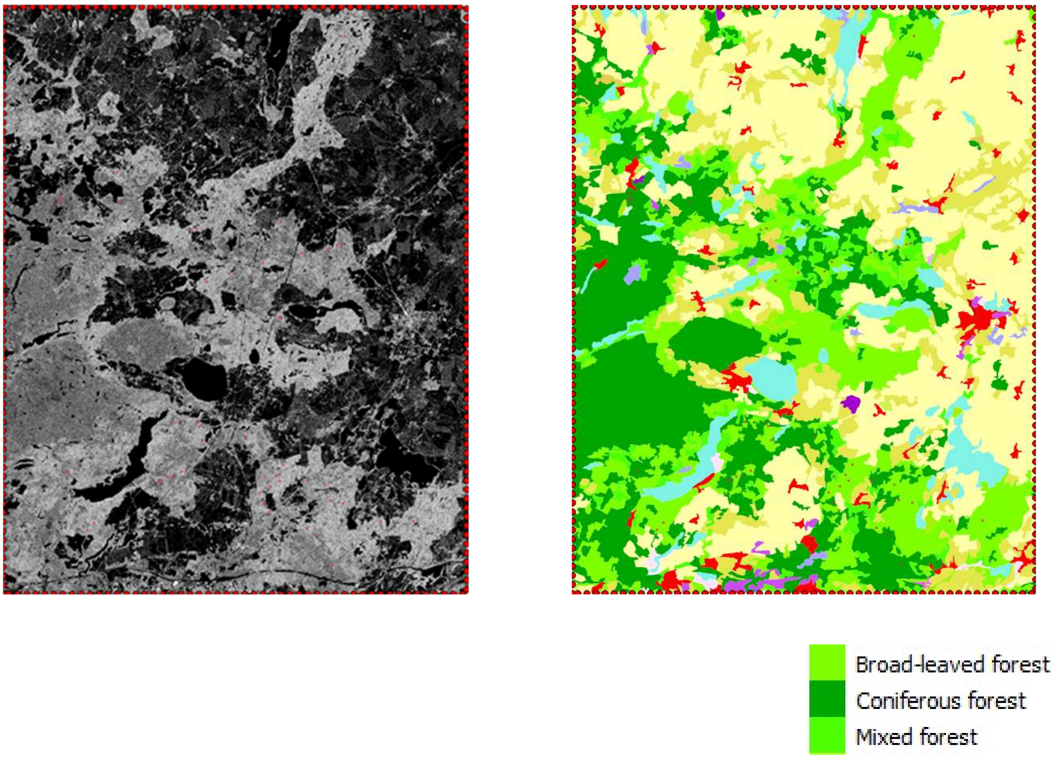

Supplementary Figure 4 Visual comparison of the winter VH and the Corine Land Cover Map of the Schorfheide-Chorin area. Among the 40 explored metrics of the radar data, the yearly and winter VH (VH_{year} and VH_{winter}) best described forest maturity, consistent with previous studies showing the better discriminatory ability of VH for forest areas and biomass[1,2]. The ability of both variables to describe the forest area was also determined in a visual comparison of backscatter intensity and the Corine Land Cover map. The Corine Land Cover map was accessed on 23.08.2017 from https://land.copernicus.eu/pan-european/corine-land-cover.

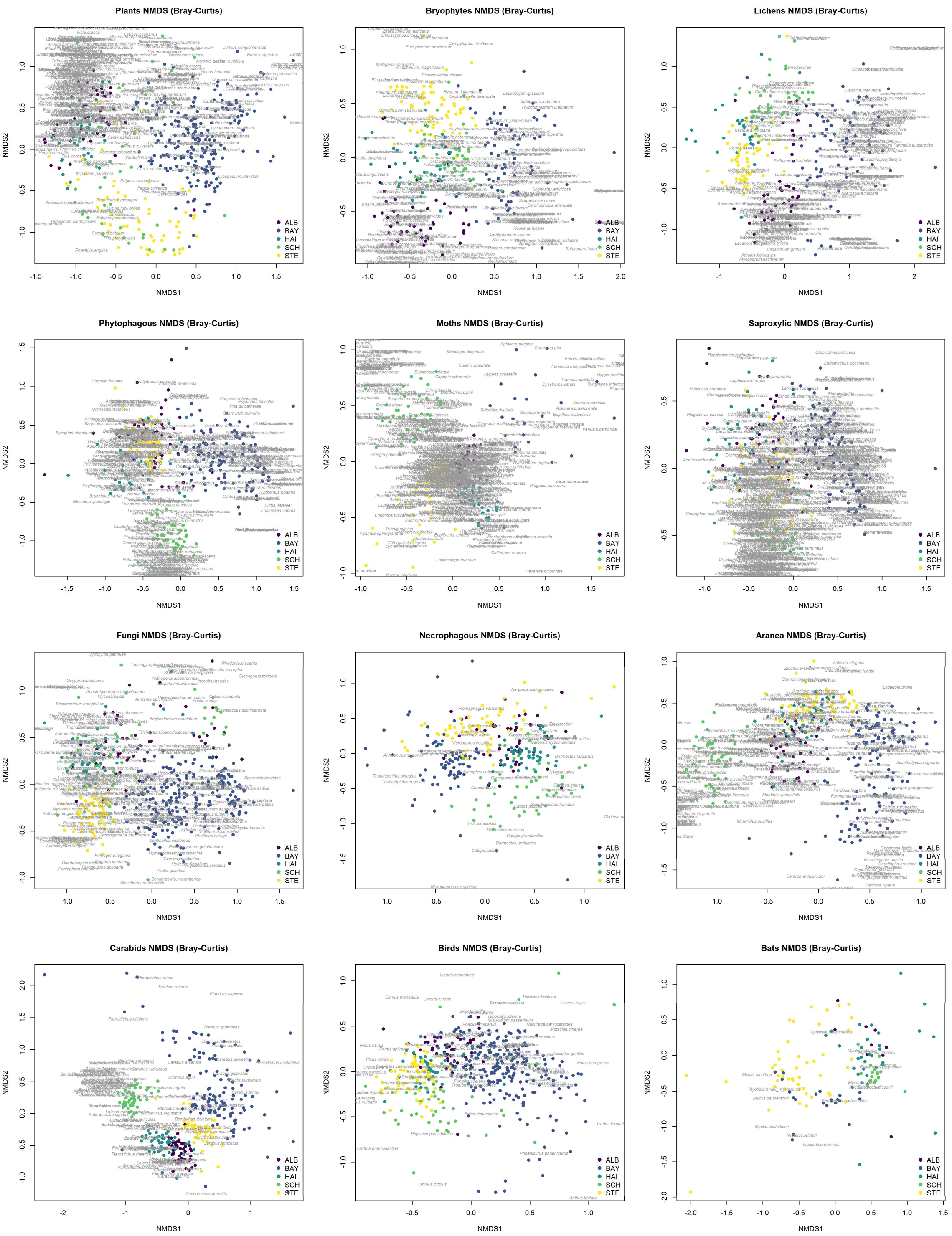

Supplementary Figure 5 Non-metric multidimensional scaling (NMDS) of 12 functional groups. Source data are provided as a Source Data file.

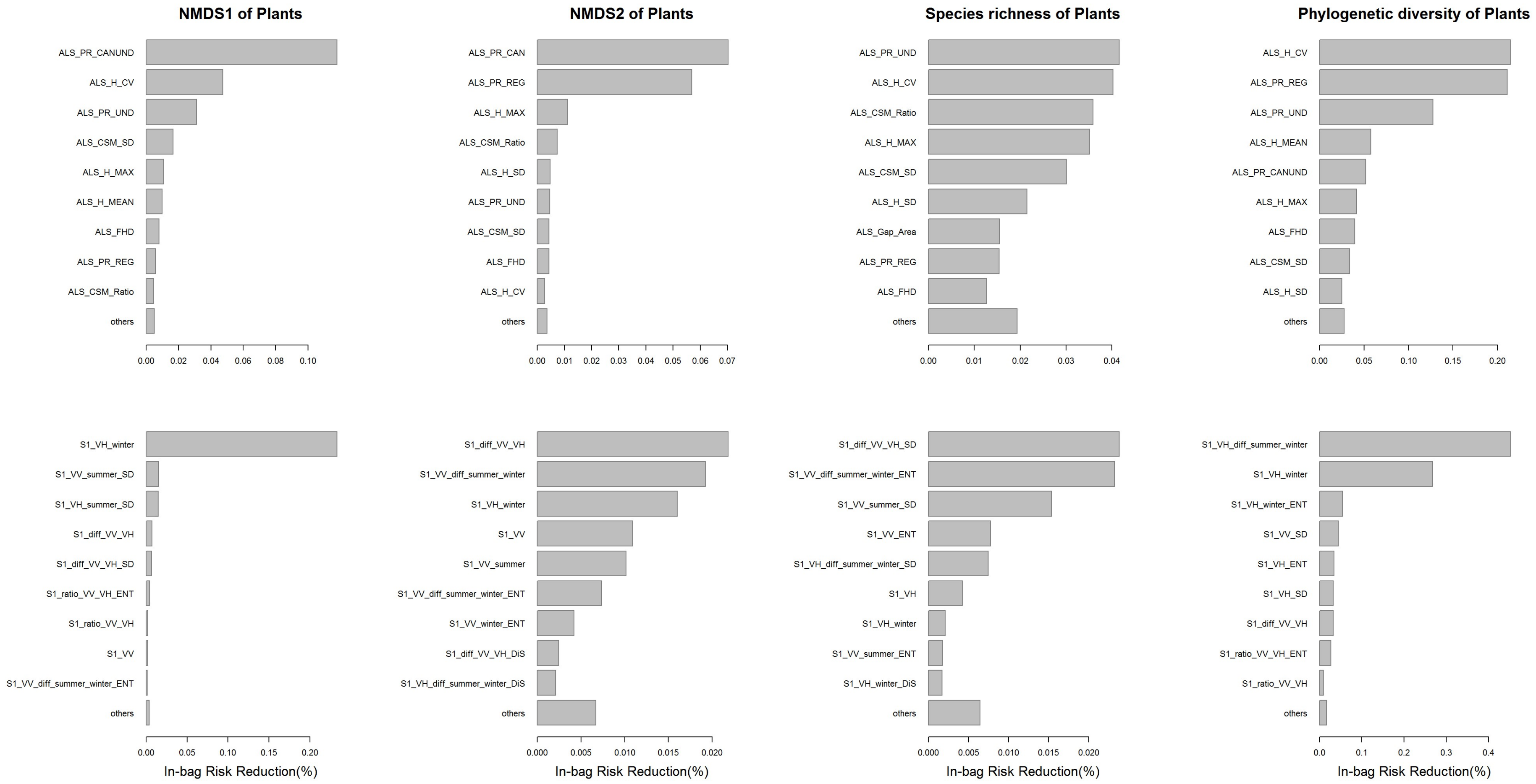

Supplementary Figure 6 Importance of the variables in the assemblage habitat models (boosted generalised additive models, GAMs) of vascular plants according to the ALS (first row) and radar (second row) data sets

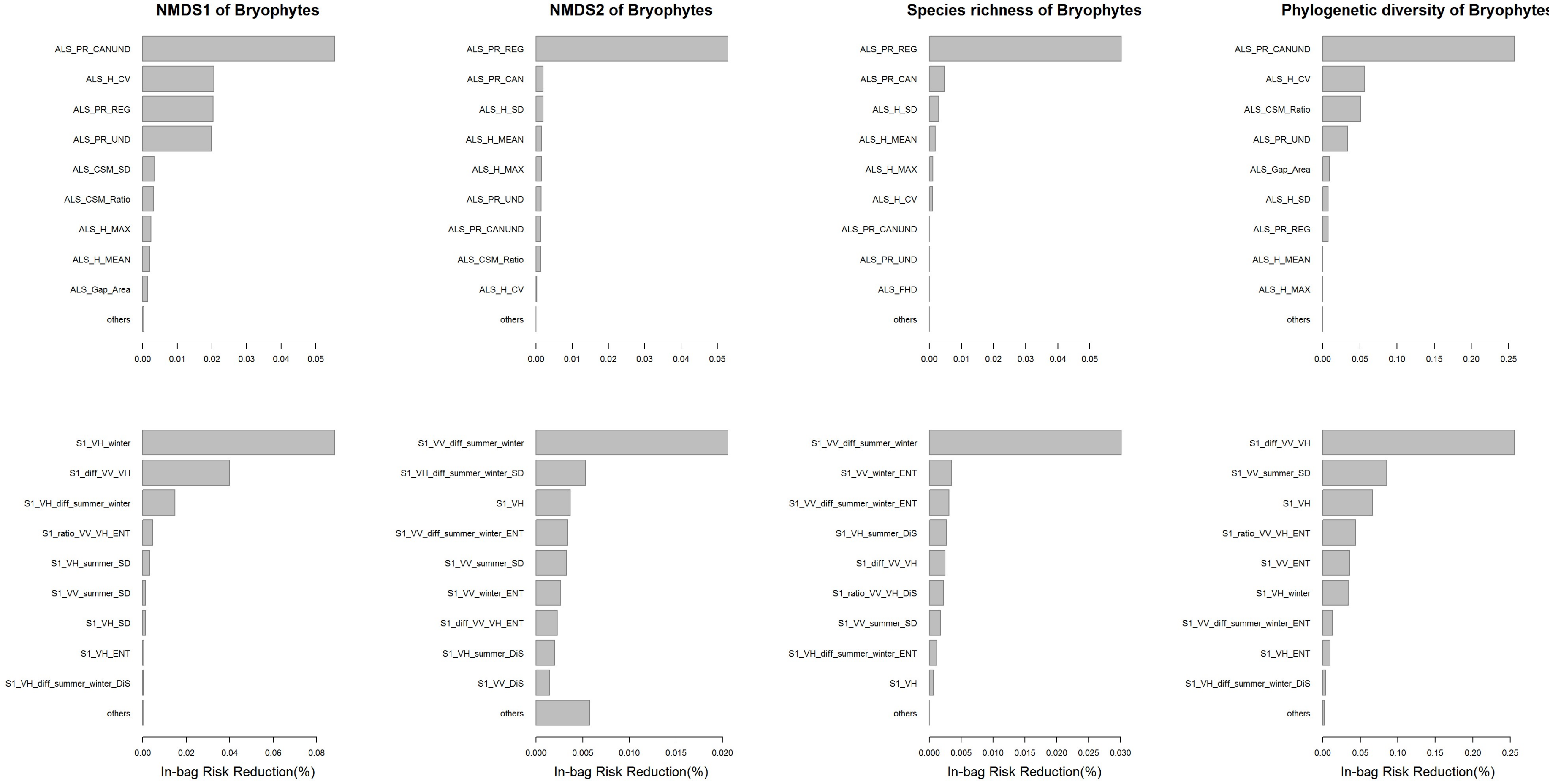

Supplementary Figure 7 Importance of the variables in the assemblage habitat models (boosted GAMs) of bryophytes according to the ALS (first row) and radar (second row) data sets

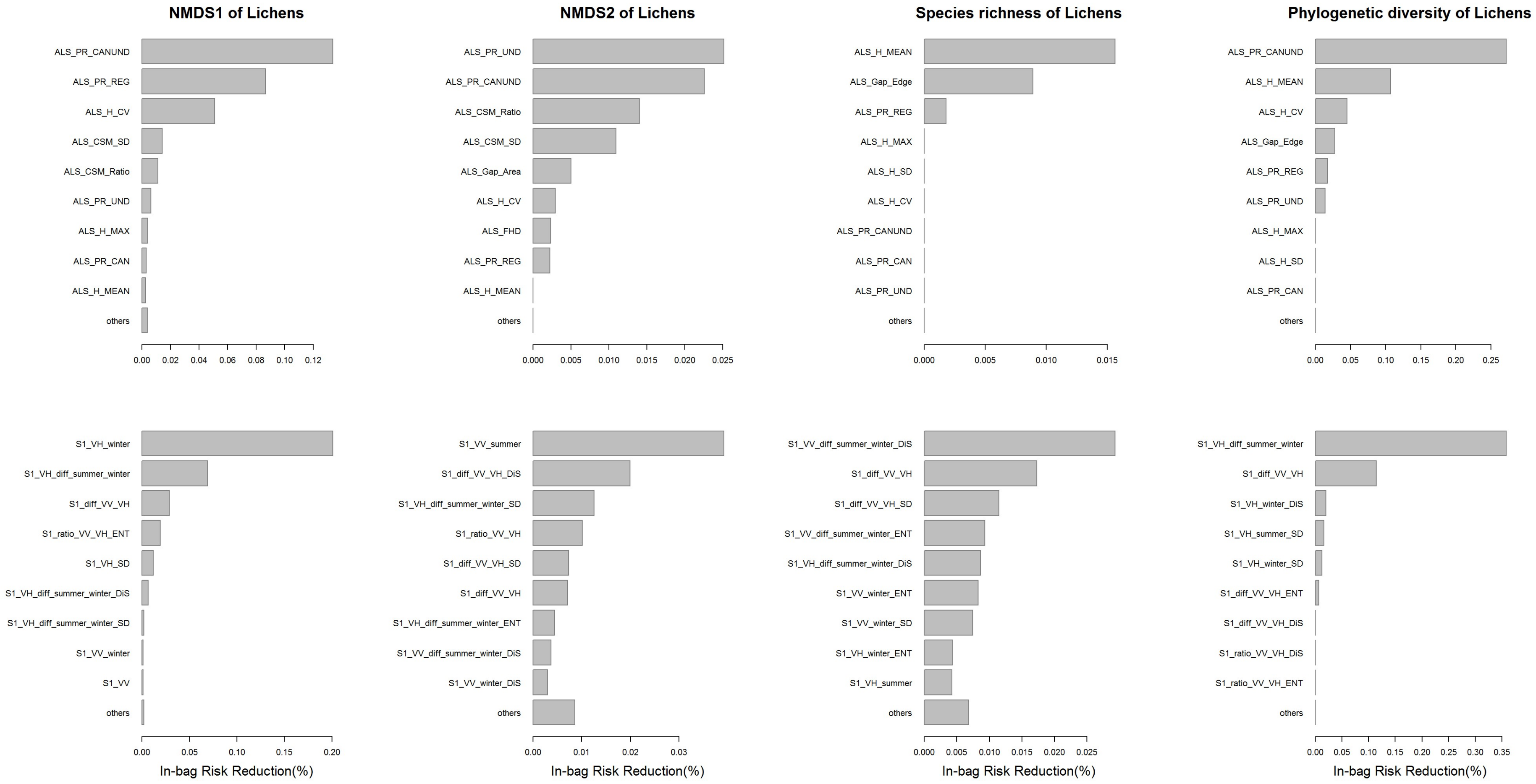

Supplementary Figure 8 Importance of the variables in the assemblage habitat models (boosted GAMs) of lichens according to the ALS (first row) and radar (second row) data sets

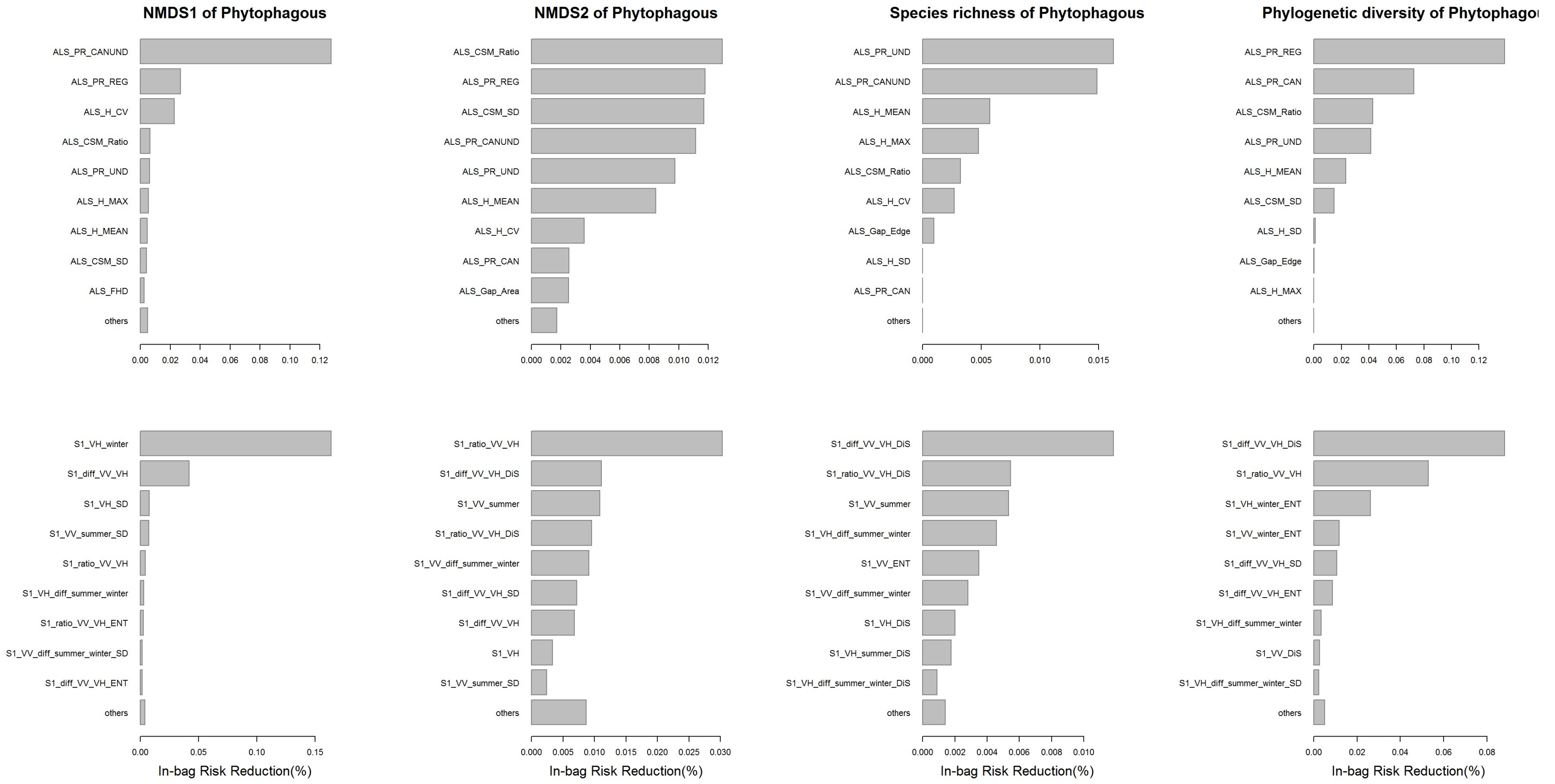

Supplementary Figure 9 Importance of the variables in the assemblage habitat models (boosted GAMs) of phytophagous beetles according to the ALS (first row) and radar (second row) data sets

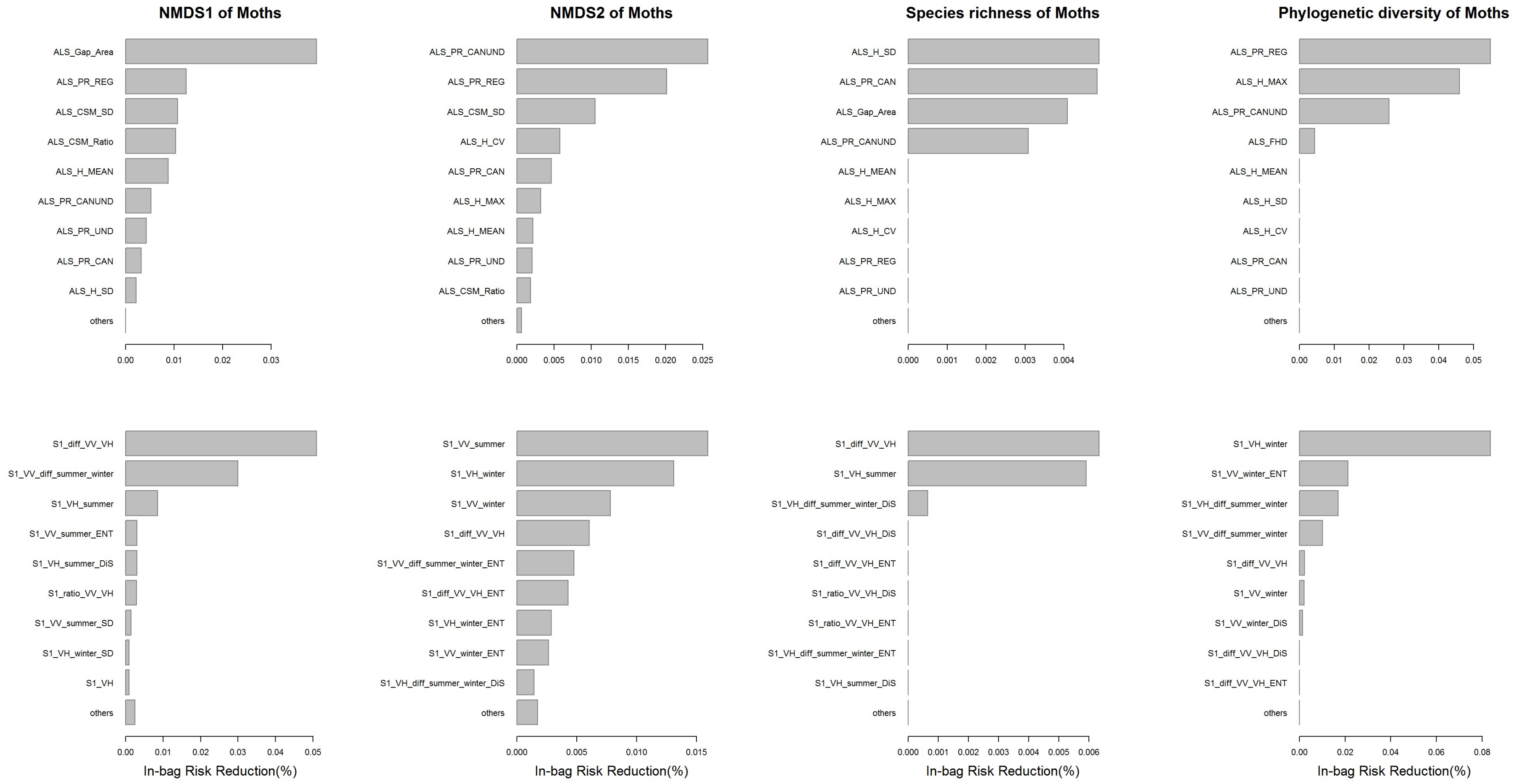

Supplementary Figure 10 Importance of the variables in the assemblage habitat models (boosted GAMs) of moths according to the ALS (first row) and radar (second row) data sets

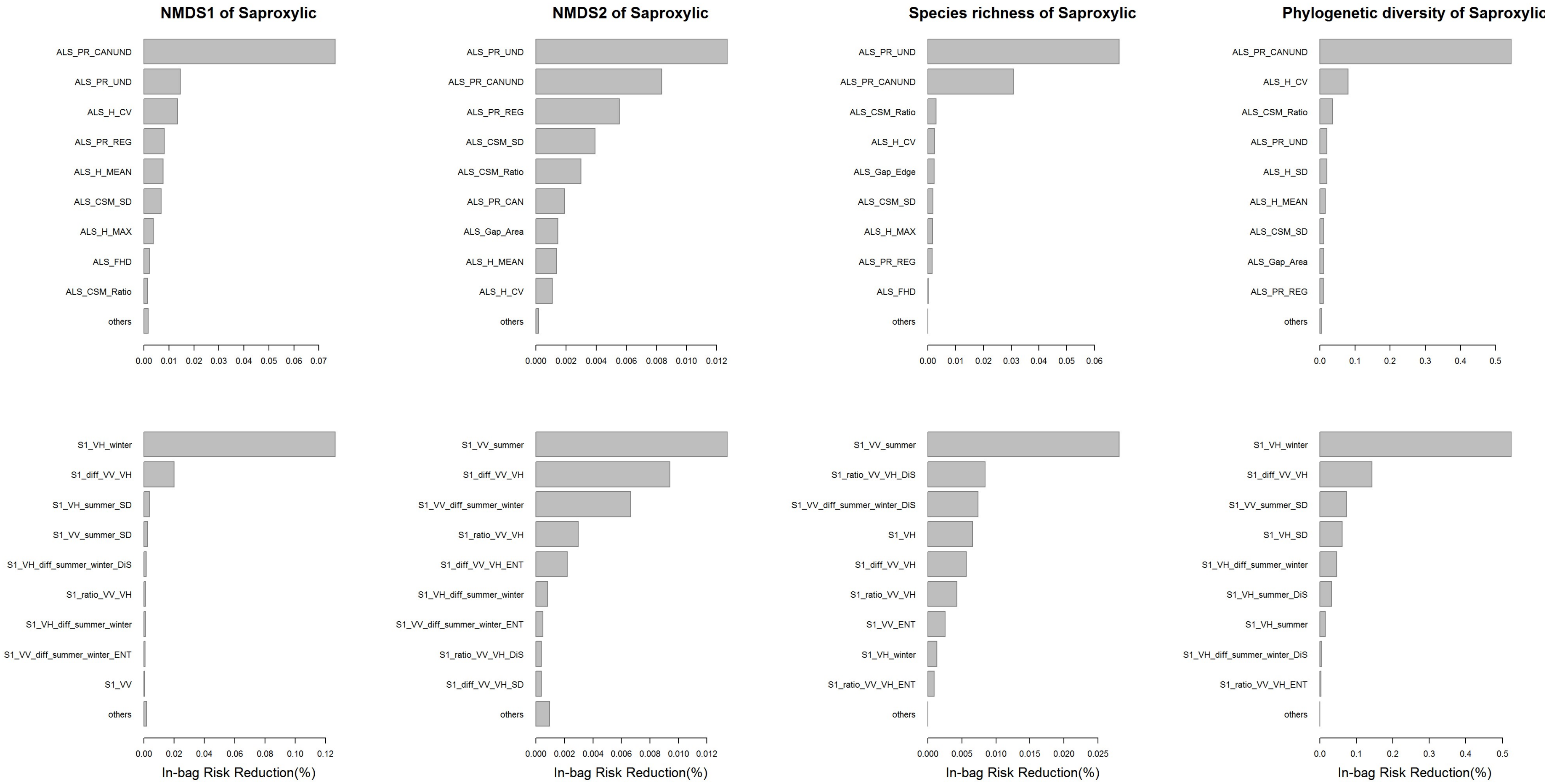

Supplementary Figure 11 Importance of the variables in the assemblage habitat models (boosted GAMs) of saproxylic beetles according to the ALS (first row) and radar (second row) data sets

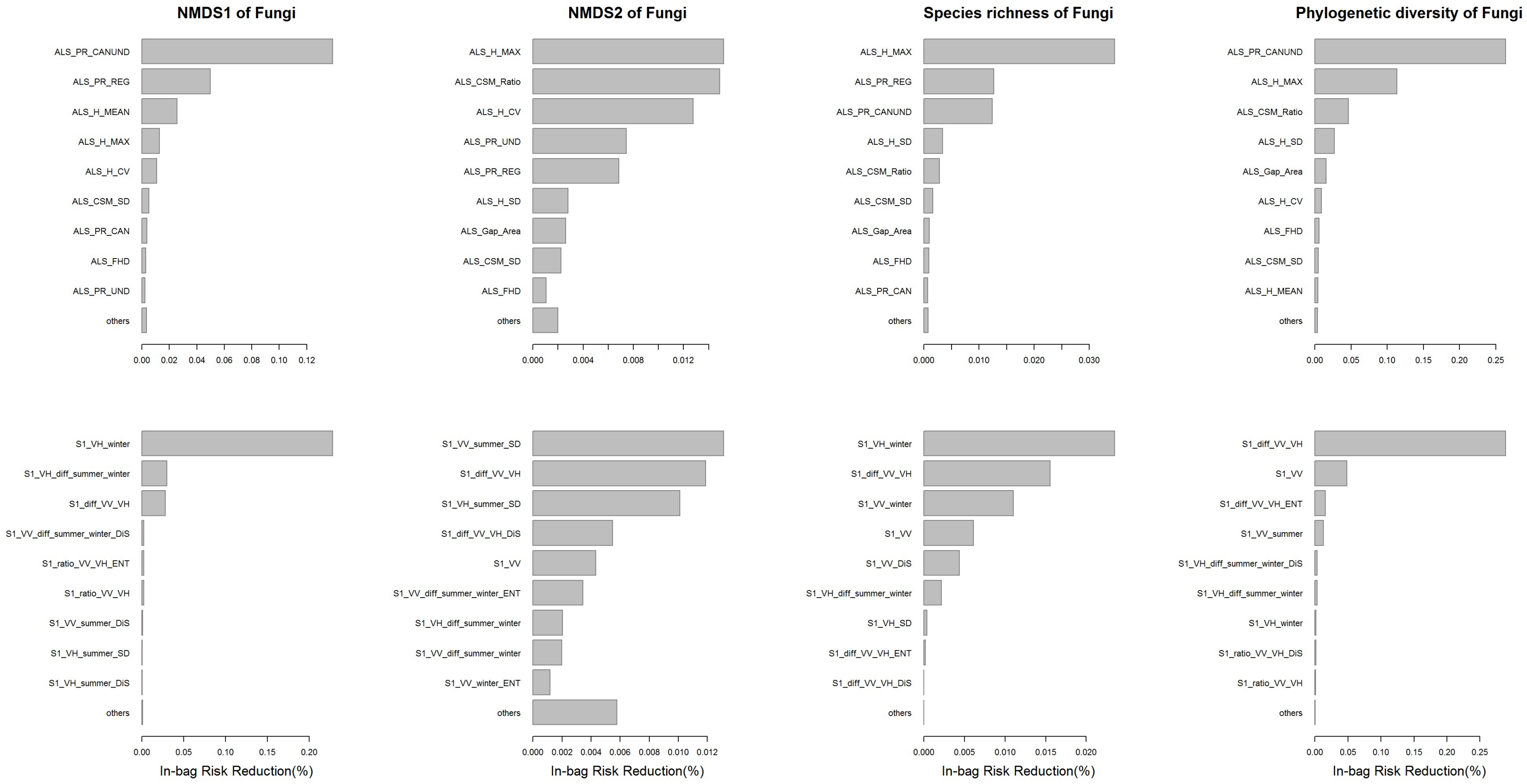

Supplementary Figure 12 Importance of the variables in the assemblage habitat models (boosted GAMs) of fungi according to the ALS (first row) and radar (second row) data sets

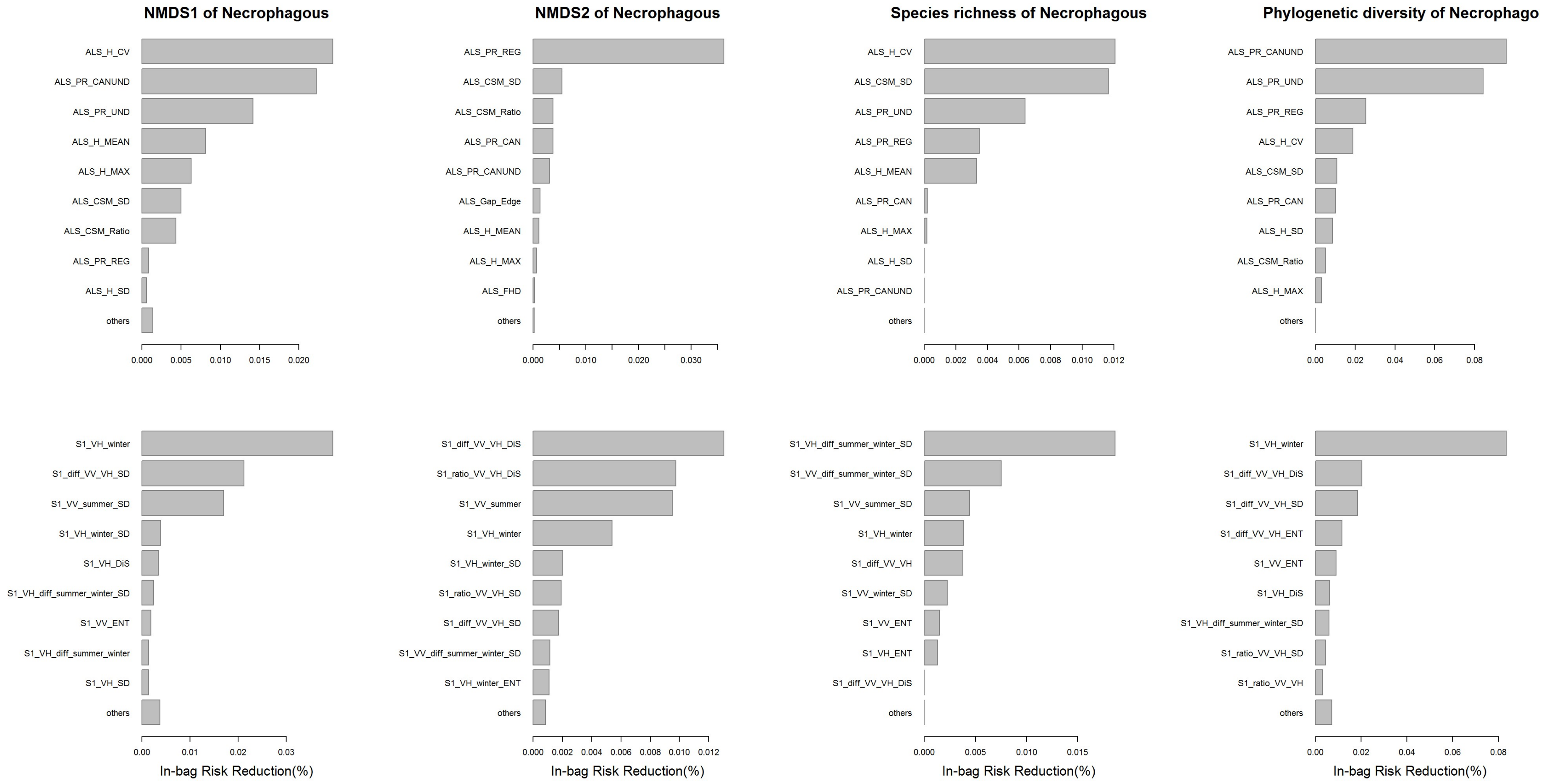

Supplementary Figure 13 Importance of the variables in the assemblage habitat models (boosted GAMs) of necrophagous beetles according to the ALS (first row) and radar (second row) data sets

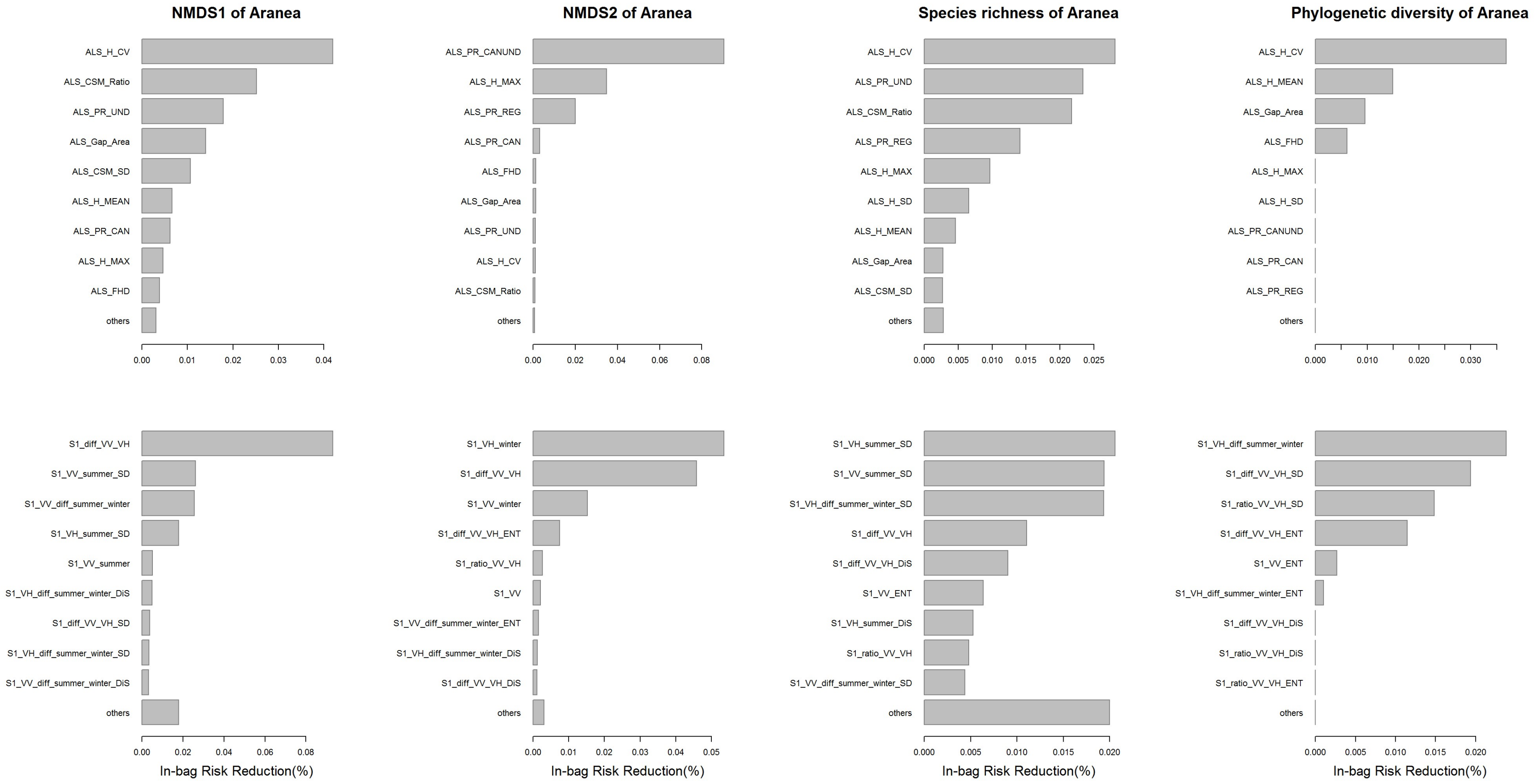

Supplementary Figure 14 Importance of the variables in the assemblage habitat models (boosted GAMs) of spiders according to the ALS (first row) and radar (second row) data sets

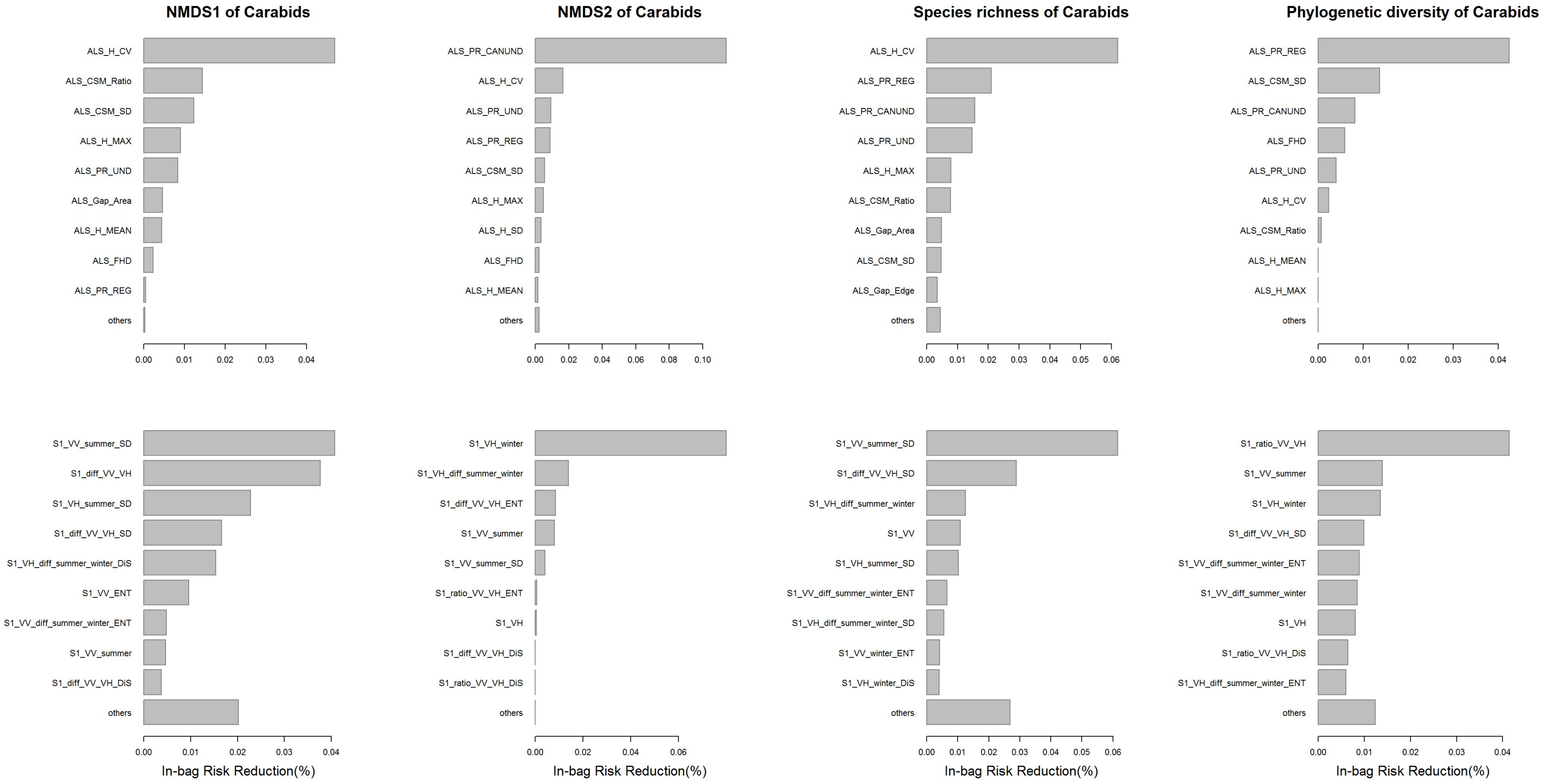

Supplementary Figure 15 Importance of the variables in the assemblage habitat models (boosted GAMs) of carabid beetles according to the ALS (first row) and radar (second row) data sets

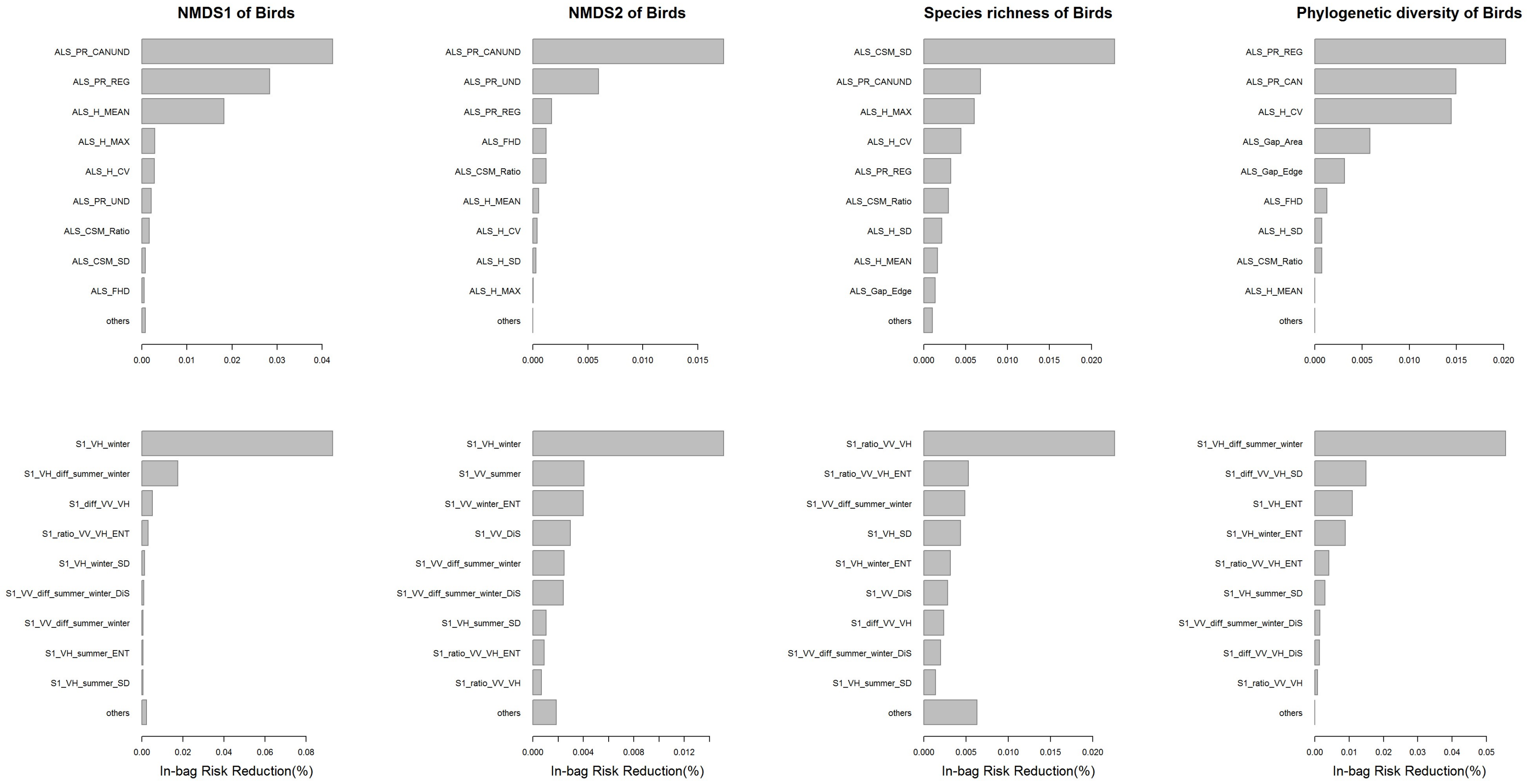

Supplementary Figure 16 Importance of the variables in the assemblage habitat models (boosted GAMs) of birds according to the ALS (first row) and radar (second row) data sets

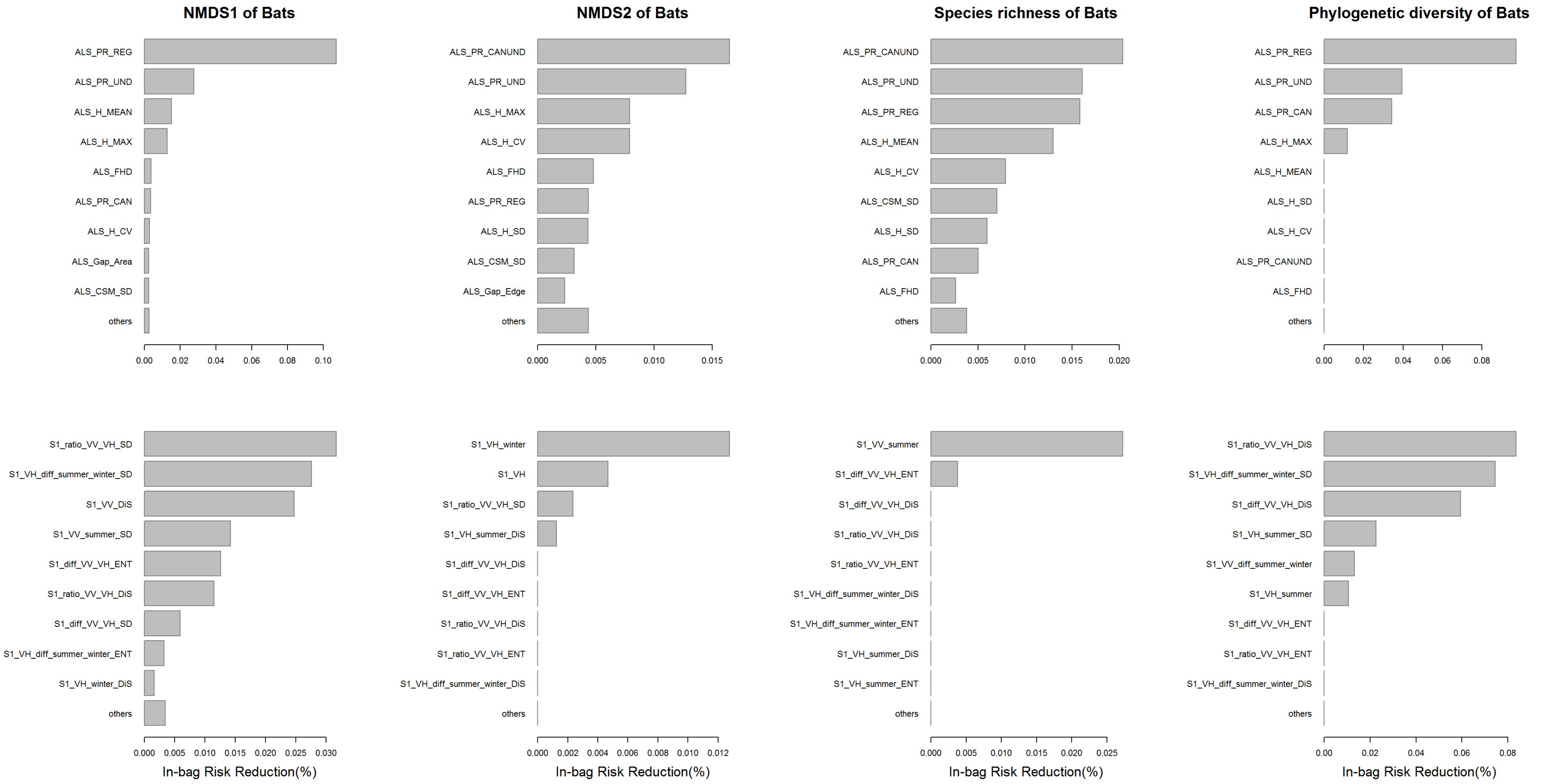

Supplementary Figure 17 Importance of the variables in the assemblage habitat models (boosted GAMs) of bats according to the ALS (first row) and radar (second row) data sets

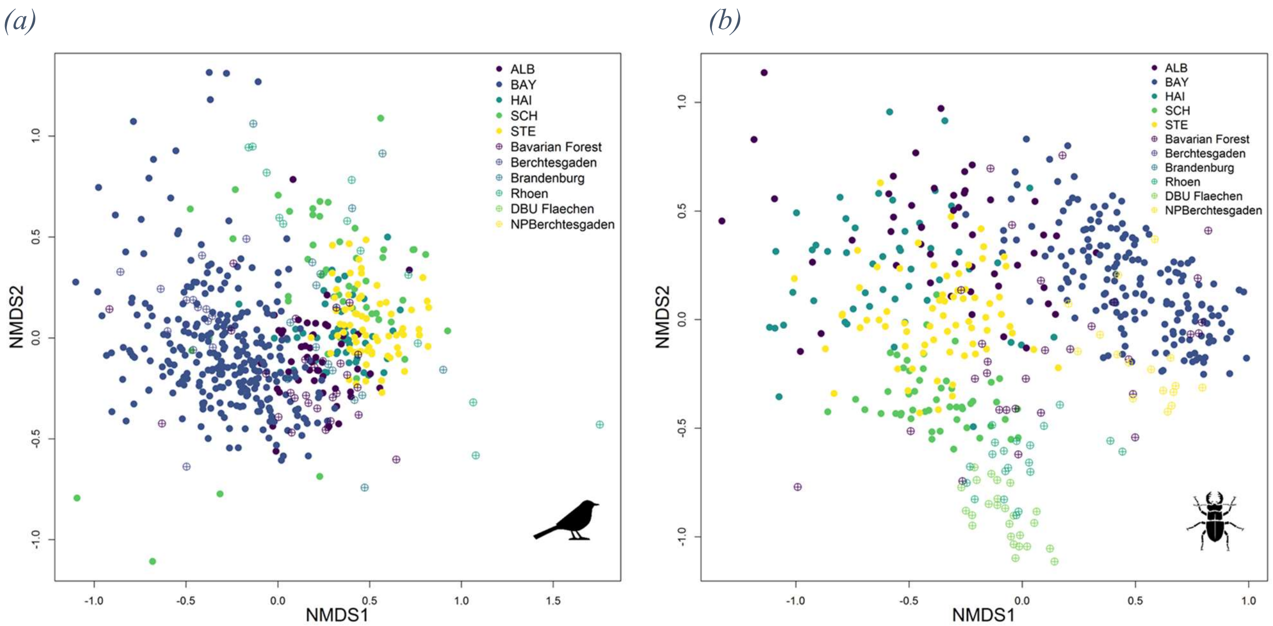

Supplementary Figure 18 The assemblage composition of (a) birds and (b) saproxylic beetles in the different sampling regions as evaluated by NMDS. The first five regions were training regions, and the remainder external validating regions, located outside the training regions. Source data are provided as a Source Data file.

(a) *(b)*

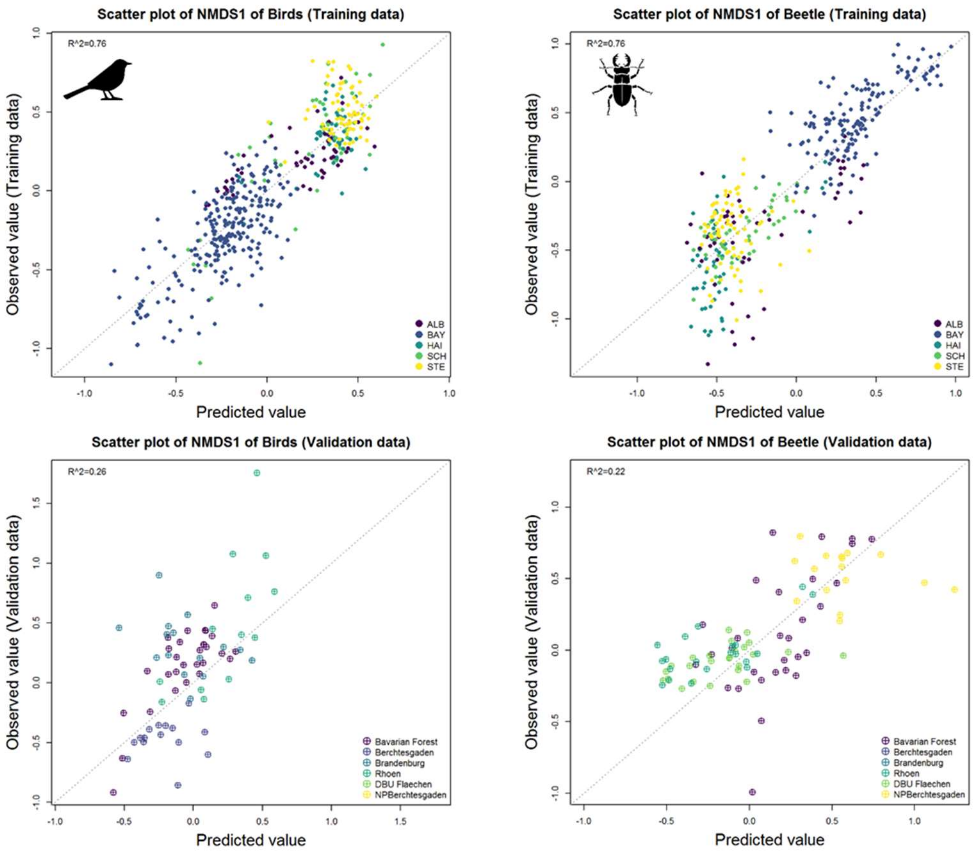

Supplementary Figure 19 Scatter plots of the observed vs. the predicted values from the training and validation data of (a) birds and (b) saproxylic beetles. Source data are provided as a Source Data file.

(a)

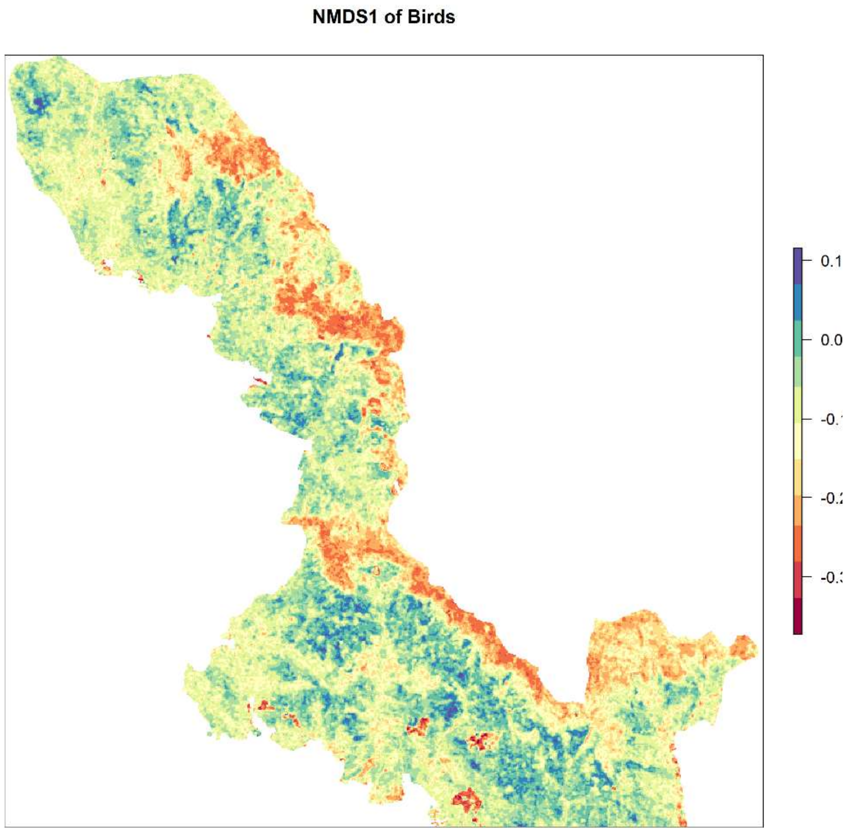

(b)

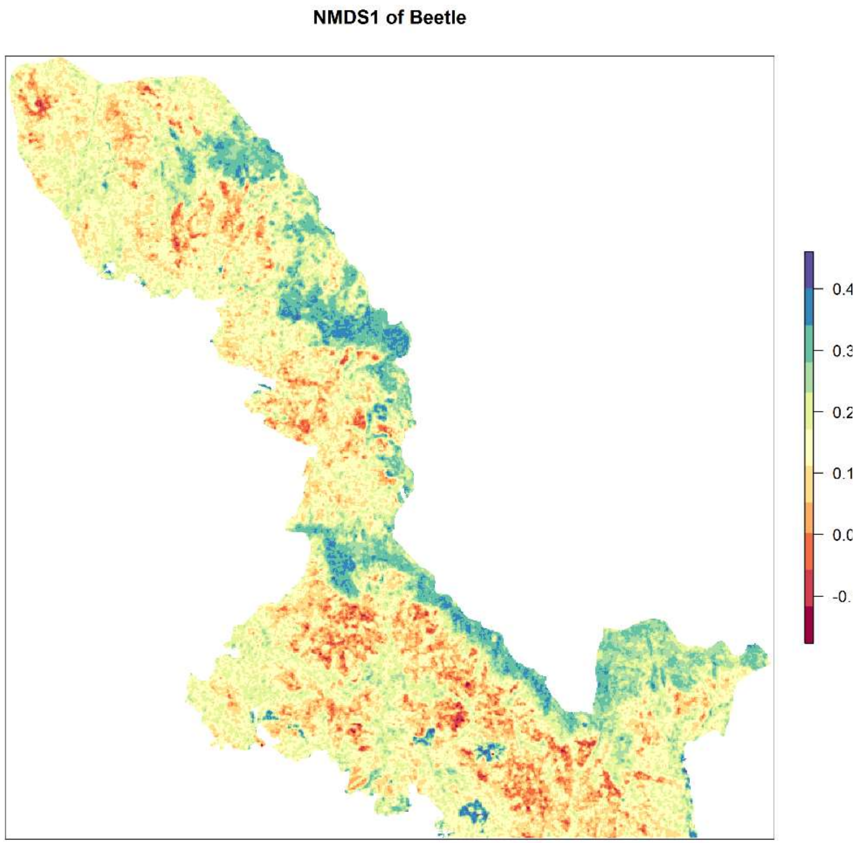

Supplementary Figure 20 Predicted maps of the Bavarian Forest National Park.

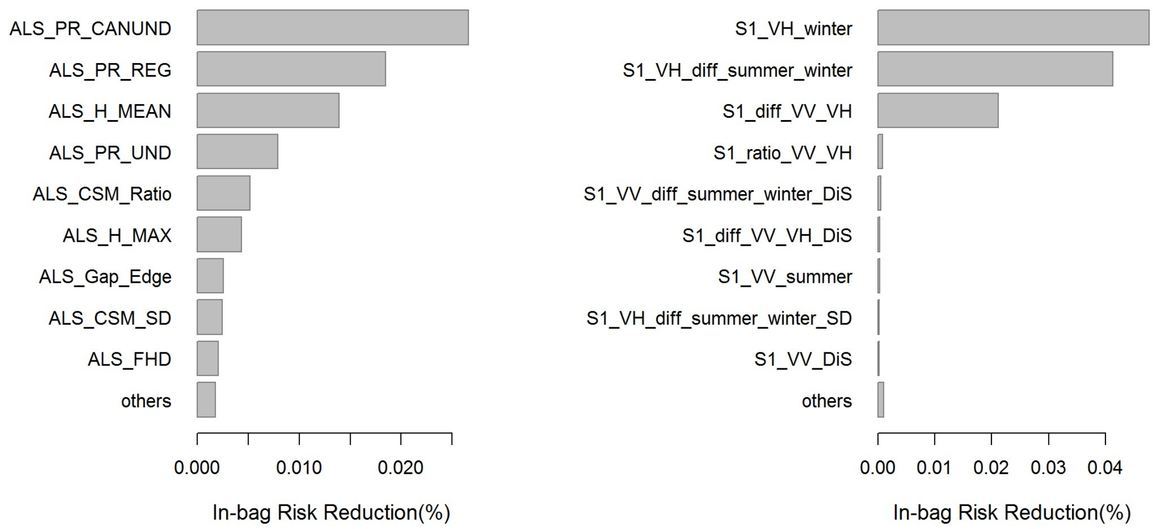

Supplementary Figure 21 Importance of the variables in the coniferous ratio models (boosted GAMs) according to the ALS (first row) and radar (second row) data sets. In a supplementary analysis, the penetration ratio of the canopy-understorey layers ($PR_{>2m}$) and the winter VH (VH_{winter}) also dominated in terms of their importance in the ALS- and radar-based models, respectively, predicting the conifer tree ratio. However, the performance of the radar-based model (Pearson's r^2 of the observed vs. the predicted conifer ratio = 0.88, $p < 0.001$) was better than that of the ALS-based model (0.66, $p < 0.001$). Despite the demonstrated potential of multi-temporal ALS in the classification of tree species, the high costs and efforts required for multiple ALS acquisitions and data processing limit the availability of multi-temporal ALS data[44]. However, given the accessibility and attributes of radar backscatters reflecting both forest maturity and tree composition, two of the main drivers of local species turnover, and heterogeneity, the use of open-access, radar-based remote sensing data offers a promising approach to modelling the biodiversity of different functional and taxonomic groups.

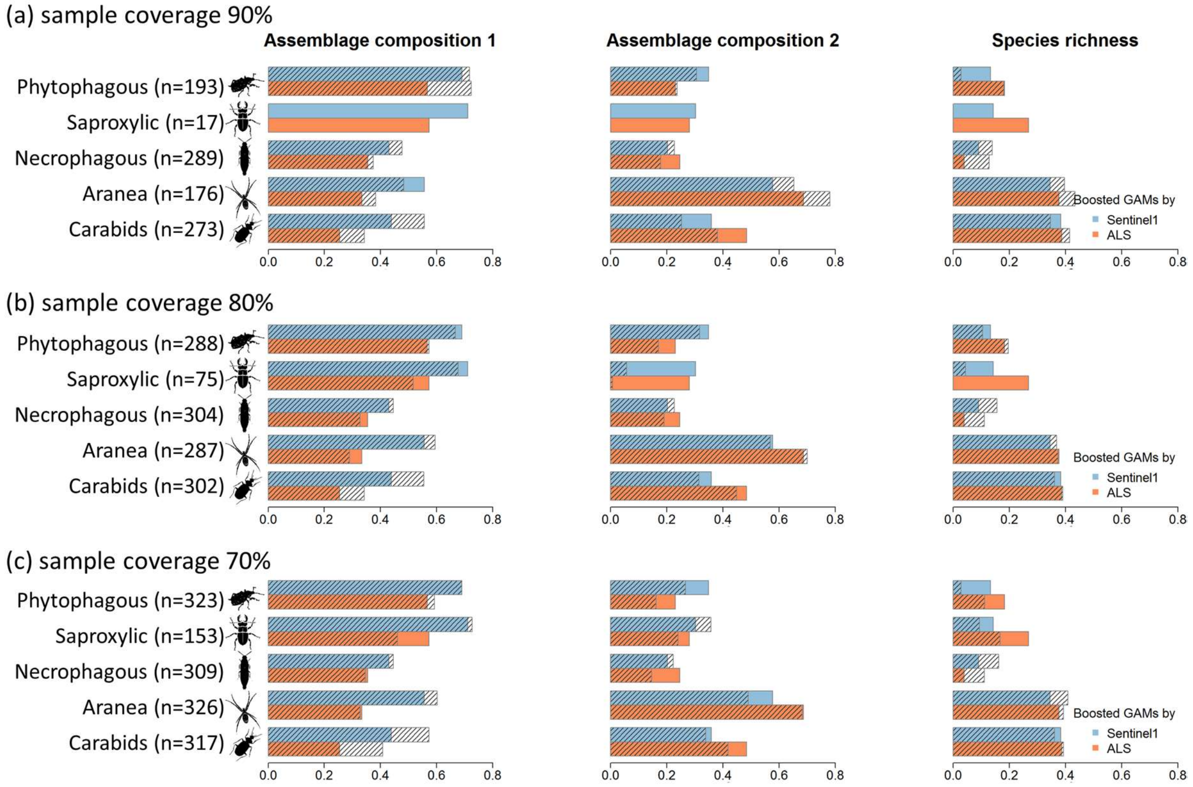

Supplementary Figure 22 Robustness test of assemblage habitat models (boosted generalised additive models) using the ALS (orange bars) and radar (blue bars) data sets against different subsets of plots in comparison to total data. The shaded bars represent R^2 (coefficient of determination) derived from the models with a subset of plots over (a) sample coverage 90%, (b) sample coverage 80%, (c) sample coverage 70% using the method by Chao and Jost [3] to calculate sample completeness for each sample based on the individuals with function iNext in the add-on package iNext in R. As the number of plots over sample coverage 90% of saproxylic beetle is too low for cross validation, we excluded this analysis. Note that all analyses matched very well the finding of our total data, underlying the sufficient data of our original analyses (Fig. 3).

Supplementary Tables

Supplementary Table 1 Radar metrics

	Variables	Description
*Mean backscatter**		
VH	VH_{year}	Yearly median of VH polarisation
	VH_{winter}	Winter median of VH polarisation
	VH_{summer}	Summer median of VH polarisation
	$VH_{\text{s-w}}$	Difference between VH_{winter} and VH_{summer}
VV	VV_{year}	Yearly median of VV polarisation
	VV_{winter}	Winter median of VV polarisation
	VV_{summer}	Summer median of VV polarisation
	$VV_{\text{s-w}}$	Difference between VV_{winter} and VV_{summer}
VH and VV	$VV - VH$	Difference between VH_{year} and VV_{year}
	VV/VH	Ratio of VH_{year} and VV_{year}
*Heterogeneity indices**		
VH	$VH_{\text{year(DiS)}}$	Dissimilarity of VH_{year} with neighbourhood pixels
	$VH_{\text{year(ENT)}}$	Entropy of VH_{year} with neighbourhood pixels
	$VH_{\text{year(SD)}}$	Standard deviation of VH_{year} in a 1-ha plot
	$VH_{\text{winter(DiS)}}$	Dissimilarity of VH_{winter} with neighbourhood pixels
	$VH_{\text{winter(ENT)}}$	Entropy of VH_{winter} with neighbourhood pixels
	$VH_{\text{winter(SD)}}$	Standard deviation of VH_{winter} in a 1-ha plot
	$VH_{\text{summer(DiS)}}$	Dissimilarity of VH_{summer} with neighbourhood pixels
	$VH_{\text{summer(ENT)}}$	Entropy of VH_{summer} with neighbourhood pixels
	$VH_{\text{summer(SD)}}$	Standard deviation of VH_{summer} in a 1-ha plot
	$VH_{\text{s-w(DiS)}}$	Dissimilarity of $VH_{\text{s-w}}$ with neighbourhood pixels
	$VH_{\text{s-w(ENT)}}$	Entropy of $VH_{\text{s-w}}$ with neighbourhood pixels
	$VH_{\text{s-w(SD)}}$	Standard deviation of $VH_{\text{s-w}}$ in a 1-ha plot
VV	$VV_{\text{year(DiS)}}$	Dissimilarity of VV_{year} with neighbourhood pixels
	$VV_{\text{year(ENT)}}$	Entropy of VV_{year} with neighbourhood pixels
	$VV_{\text{year(SD)}}$	Standard deviation of VV_{year} in a 1-ha plot
	$VV_{\text{winter(DiS)}}$	Dissimilarity of VV_{winter} with neighbourhood pixels
	$VV_{\text{winter(ENT)}}$	Entropy of VV_{winter} with neighbourhood pixels
	$VV_{\text{winter(SD)}}$	Standard deviation of VV_{winter} in a 1-ha plot
	$VV_{\text{summer(DiS)}}$	Dissimilarity of VV_{summer} with neighbourhood pixels
	$VV_{\text{summer(ENT)}}$	Entropy of VV_{summer} with neighbourhood pixels
	$VV_{\text{summer(SD)}}$	Standard deviation of VV_{summer} in a 1-ha plot
	$VV_{\text{s-w(DiS)}}$	Dissimilarity of $VV_{\text{s-w}}$ with neighbourhood pixels
	$VV_{\text{s-w(ENT)}}$	Entropy of $VV_{\text{s-w}}$ with neighbourhood pixels
	$VV_{\text{s-w(SD)}}$	Standard deviation of $VV_{\text{s-w}}$ in a 1 ha plot
VH and VV	$VV - VH_{\text{(DiS)}}$	Dissimilarity of *VV-VH* with neighbourhood pixels
	$VV - VH_{\text{(ENT)}}$	Entropy of *VV-VH* with neighbourhood pixels
	$VV - VH_{\text{(SD)}}$	Standard deviation of *VV-VH* in a 1-ha plot
	$VV/VH_{\text{(DiS)}}$	Dissimilarity of *VV/VH* with neighbourhood pixels
	$VV/VH_{\text{(ENT)}}$	Entropy of *VV/VH* with neighbourhood pixels
	$VV/VH_{\text{(SD)}}$	Standard deviation of *VV/VH* in a 1-ha plot

** Mean values are based on the averages within the 1-ha plots.*
*** Dissimilarity and entropy were calculated based on a window size of 9 × 9 pixels, i.e. 0.81 ha, to consider the contrast and orderliness of the corresponding metrics within each plot*

Supplementary Table 2 ALS data sources

Region	Sensor	Year	Month	Flight height (m, agl)	Pulse density* (pls/m²)	Data provider
HAI	Riegl Q560	2008	August	400–600	8.2	Max-Planck-Institute for Biogeochemistry, Jena
ALB	Riegl Q560	2010	July	400–600	21.09	
SCH	Riegl Q560	2009	September	400–600	27.03	
STE	Riegl Q560/ VQ780i	2015 / 2018	September /Mai	650–700	40.07	Munich University of Applied Sciences
BAY	Riegl Q560	2007	Mai	350	33.25	Bavarian Forest National Park

** The pulse density was derived from the clipped point cloud per plot and aggregated as a median value for each region.*

Supplementary Table 3 ALS metrics

Variable	Description
H_{mean}	Mean height of vegetation returns
H_{max}	Maximum height of vegetation returns
H_{SD}	Standard deviation of the height of vegetation returns
H_{CV}	Coefficient of variation of the height of vegetation returns
$PR_{\text{h}>5m}$	Penetration ratio of canopy layer (>5 m above ground)
$PR_{5m>h>2m}$	Penetration ratio of the understorey (2–5 m)
$PR_{2m>h>0m}$	Penetration ratio of the regeneration layer (below 2 m)
$PR_{\text{h}>2m}$	Penetration ratio of the canopy-understorey layers (above 2 m)
FHD	Foliage height diversity*
Gap_{Edge}	Total edge length of gaps (square-root-transformed)
Gap_{Area}	Total area of gaps (square-root-transformed)
CSM_{Ratio}	Ratio between the values of the canopy surface and flat areas
CSM_{SD}	Standard deviation of the canopy surface height

** Foliage height diversity was calculated for the canopy, understorey and regeneration layers.*

Supplementary Table 4 Summary of the results of the canonical correlation analysis. The first and second axes showed the highest canonical correlation coefficients (the correlation between the pairs of canonical axes from the two datasets), 0.92 and 0.75, and explained 54.01% and 13.18% of the variance respectively. The canonical correlation coefficients of the third and fourth axes were also high, 0.68 and 0.62 respectively, and explained 8.64% and 6.41% of the variance, with the cumulative variance of the first four axes accounting for 82.24% of the total variance.

Canonical correlation pair	Canonical correlation	Eigenvalue	Variance (%)	Cumulative variance (%)	Statistic value	*F* value	Num *df*	Den *df*	*p* value
1	0.92	5.30	54.01	54.01	0.00489	5.29	520	5126.7	< 0.0001
2	0.75	1.29	13.18	67.19	0.03085	3.60	468	4763.6	< 0.0001
3	0.68	0.85	8.64	75.83	0.07077	2.99	418	4395.6	<0.0001
4	0.62	0.63	6.41	82.24	0.13082	2.54	370	4022.4	< 0.0001
5	0.57	0.48	4.89	87.14	0.21322	2.17	324	3643.9	< 0.0001
6	0.48	0.30	3.09	90.23	0.31562	1.85	280	3260	< 0.0001
7	0.44	0.25	2.51	92.73	0.41144	1.66	238	2870.5	< 0.0001
8	0.40	0.19	1.90	94.64	0.51272	1.49	198	2475.5	< 0.0001
9	0.38	0.16	1.67	96.30	0.60856	1.37	160	2074.9	< 0.001
10	0.35	0.14	1.43	97.74	0.70812	1.22	124	1668.9	0.0555
11	0.31	0.11	1.11	98.84	0.80781	1.03	90	1257.8	0.3988
12	0.24	0.06	0.64	99.48	0.89549	0.82	58	842	0.8226
13	0.22	0.05	0.52	100	0.95167	0.77	28	422	0.8021

Supplementary Table 5 Pearson's correlation coefficient for the ALS metrics vs. axes 1–9 of the canonical correlation analysis (Ycan1–9). We indicated the significant values (p < 0.05) with black numbers.

	Ycan1	Ycan2	Ycan3	Ycan4	Ycan5	Ycan6	Ycan7	Ycan8	Ycan9
H_{mean}	−0.70	0.09	−0.19	0.01	−0.08	0.14	−0.63	0.05	0.04
H_{max}	−0.71	0.35	0.14	−0.05	0.19	−0.01	−0.47	−0.17	−0.04
H_{SD}	−0.40	0.58	0.13	−0.29	0.43	−0.04	−0.19	−0.16	0.03
H_{CV}	0.28	0.44	0.30	−0.35	0.30	−0.20	0.41	−0.16	−0.19
$PR_{h>2m}$	−0.94	−0.18	0.00	0.16	0.03	−0.03	−0.07	−0.13	0.07
$PR_{h>5m}$	−0.89	−0.18	−0.03	0.21	0.03	0.02	−0.22	−0.12	0.04
$PR_{2m>h>0m}$	−0.33	0.36	−0.45	−0.27	0.54	−0.07	0.04	0.25	−0.19
$PR_{5m>h>2m}$	−0.35	0.18	−0.09	−0.27	0.13	−0.37	0.55	−0.39	0.23
FHD	−0.10	0.45	0.04	−0.46	0.46	0.13	0.51	0.00	0.01
Gap_{Edge}	0.65	0.15	0.33	−0.31	−0.04	−0.23	0.22	0.33	0.07
Gap_{Area}	0.81	0.30	0.19	−0.16	−0.08	−0.16	0.15	0.28	0.02
CSM_{Ratio}	−0.08	0.16	0.22	−0.14	0.29	0.18	−0.36	−0.12	−0.07
CSM_{SD}	−0.39	0.53	0.32	−0.30	0.00	0.06	−0.32	−0.11	−0.10

Supplementary Table 6 Pearson's correlation coefficient for the radar metrics vs. axes 1–9 of the canonical correlation analysis (Xcan 1–9). We indicated the significant values ($p < 0.05$) with black numbers.

	Xcan1	Xcan2	Xcan3	Xcan4	Xcan5	Xcan6	Xcan7	Xcan8	Xcan9
VH_{year}	−0.89	−0.09	−0.10	−0.30	−0.02	−0.02	0.13	−0.11	0.04
VH_{winter}	−0.88	−0.06	−0.25	−0.26	−0.04	−0.06	0.13	−0.02	0.01
VH_{summer}	−0.79	−0.13	0.13	−0.27	0.04	0.11	0.06	−0.24	0.00
$VH_{\mathrm{s-w}}$	0.72	−0.01	0.47	0.19	0.09	0.17	−0.15	−0.15	−0.02
VV_{year}	−0.83	0.01	0.10	−0.40	−0.10	−0.08	0.17	−0.11	−0.06
VV_{winter}	−0.84	0.05	−0.11	−0.39	−0.07	−0.06	0.16	−0.02	−0.08
VV_{summer}	−0.80	−0.02	0.26	−0.26	−0.13	−0.02	0.15	−0.19	−0.05
$VV_{\mathrm{s-w}}$	0.34	−0.11	0.56	0.30	−0.06	0.08	−0.07	−0.24	0.07
$VV - VH$	0.60	0.24	0.41	−0.02	−0.12	−0.11	0.01	0.06	−0.20
VV/VH	0.30	−0.25	−0.40	0.38	0.22	0.16	−0.17	0.06	0.20
$VH_{\mathrm{year(DiS)}}$	−0.30	0.19	0.64	−0.02	0.38	0.10	−0.01	0.11	0.07
$VH_{\mathrm{year(ENT)}}$	−0.59	−0.20	0.57	0.08	0.18	0.12	0.15	0.02	−0.11
$VH_{\mathrm{year(SD)}}$	0.19	0.69	0.34	0.24	0.08	0.05	−0.05	0.21	0.13
$VH_{\mathrm{winter(DiS)}}$	−0.08	−0.08	0.75	0.19	0.25	0.17	0.00	0.18	0.09
$VH_{\mathrm{winter(ENT)}}$	−0.20	−0.25	0.72	0.24	0.15	0.19	0.09	0.11	0.06
$VH_{\mathrm{winter(SD)}}$	0.26	0.57	0.31	0.34	0.08	0.07	0.05	0.25	0.09
$VH_{\mathrm{summer(DiS)}}$	−0.13	−0.03	0.50	0.05	0.33	0.02	−0.01	0.19	−0.01
$VH_{\mathrm{summer(ENT)}}$	−0.33	−0.36	0.35	0.14	0.21	0.03	0.09	0.08	−0.14
$VH_{\mathrm{summer(SD)}}$	0.22	0.67	0.34	0.15	0.12	0.04	0.00	0.26	0.07
$VH_{\mathrm{s-w(DiS)}}$	−0.20	−0.01	0.36	0.14	0.07	0.25	−0.14	−0.03	0.34
$VH_{\mathrm{s-w(ENT)}}$	−0.36	−0.04	0.35	0.14	−0.10	0.28	−0.17	0.02	0.29
$VH_{\mathrm{s-w(SD)}}$	0.15	0.31	0.17	0.09	0.30	0.01	0.25	0.11	−0.07
$VV_{\mathrm{year(DiS)}}$	−0.10	0.27	0.52	−0.06	0.44	0.08	−0.02	0.12	−0.05
$VV_{\mathrm{year(ENT)}}$	−0.43	−0.15	0.46	−0.01	0.29	0.10	0.13	−0.02	−0.19
$VV_{\mathrm{year(SD)}}$	0.20	0.67	0.37	0.24	0.10	0.08	−0.05	0.23	0.13
$VV_{\mathrm{winter(DiS)}}$	−0.12	−0.07	0.66	0.14	0.37	−0.02	0.10	0.02	−0.05
$VV_{\mathrm{winter(ENT)}}$	−0.28	−0.35	0.63	0.14	0.18	0.03	0.20	0.05	−0.11
$VV_{\mathrm{winter(SD)}}$	0.21	0.56	0.33	0.29	0.12	0.06	0.00	0.22	0.10
$VV_{\mathrm{summer(DiS)}}$	−0.14	0.06	0.41	0.10	0.49	−0.07	−0.03	0.20	−0.02
$VV_{\mathrm{summer(ENT)}}$	−0.38	−0.30	0.22	0.19	0.41	−0.07	0.05	0.07	−0.08
$VV_{\mathrm{summer(SD)}}$	0.26	0.68	0.32	0.19	0.10	0.11	0.02	0.24	0.17
$VV_{\mathrm{s-w(DiS)}}$	0.02	0.06	0.09	−0.22	−0.14	−0.11	0.23	−0.08	0.47
$VV_{\mathrm{s-w(ENT)}}$	−0.09	−0.01	0.12	−0.22	−0.33	0.01	0.09	−0.04	0.30
$VV_{\mathrm{s-w(SD)}}$	0.20	0.35	0.08	−0.03	0.21	−0.04	0.06	0.21	0.05
$VV - VH_{\mathrm{(DiS)}}$	−0.40	0.39	0.24	0.18	0.23	−0.23	−0.02	0.00	0.06
$VV - VH_{\mathrm{(ENT)}}$	−0.51	0.34	0.27	0.16	−0.03	−0.40	−0.02	0.01	−0.01
$VV - VH_{\mathrm{(SD)}}$	0.38	0.32	0.13	0.15	0.35	0.01	0.22	0.20	−0.05
$VV/VH_{\mathrm{(DiS)}}$	−0.21	0.33	0.03	0.14	0.34	−0.39	−0.16	0.05	−0.12
$VV/VH_{\mathrm{(ENT)}}$	−0.20	0.23	−0.03	0.14	0.02	−0.62	−0.22	−0.05	−0.19
$VV/VH_{\mathrm{(SD)}}$	0.09	0.57	0.34	0.12	0.26	0.08	0.05	0.27	0.12

Supplementary Table 7 Cross-validated performance (R^2, coefficient of determination) of the assemblage habitat models (boosted generalised additive models, GAMs) in the fixed effects models using the ALS and radar data sets.

	ALS models					Radar models				
	NMDS1	NMDS2	NMDS3	Species richness	Phylogenetic diversity	NMDS1	NMDS2	NMDS3	Species richness	Phylogenetic diversity
Plants	0.49	0.46	-	0.27	0.24	0.58	0.24	-	0.06	0.38
Bryophytes	0.39	0.32	0.08	0.16	0.23	0.55	0.17	0.17	0.09	0.31
Lichens	0.56	0.22	-	0.03	0.14	0.66	0.28	-	0.07	0.22
Phytophagous	0.57	0.23	0.28	0.18	0.27	0.69	0.35	0.16	0.13	0.14
Moths	0.37	0.41	-	0.04	0.13	0.43	0.34	-	0.01	0.17
Saproxylic	0.57	0.28	0.48	0.27	0.32	0.71	0.30	0.24	0.14	0.39
Fungi	0.62	0.36	0.11	0.39	0.41	0.78	0.27	0.05	0.36	0.34
Necrophagous	0.35	0.25	0.06	0.04	0.26	0.43	0.20	0.06	0.09	0.14
Spiders	0.33	0.69	0.37	0.38	0.05	0.56	0.58	0.14	0.34	0.03
Carabids	0.25	0.48	-	0.39	0.06	0.44	0.36	-	0.38	0.04
Birds	0.55	0.17	0.38	0.24	0.03	0.70	0.18	0.20	0.31	0.10
Bats	0.54	0.13	-	0.16	0.02	0.40	0.05	-	0.07	0.08
Median of 12 taxa	0.51	0.30	0.28	0.21	0.19	0.57	0.27	0.16	0.11	0.16

Supplementary Table 8 Cross-validated performance (root mean square error) of the assemblage habitat models (boosted GAMs) in the fixed effects models using the ALS and radar data sets.

	ALS models					Radar models				
	NMDS1	NMDS2	NMDS3	Species richness	Phylogenetic diversity	NMDS1	NMDS2	NMDS3	Species richness	Phylogenetic diversity
Plants	0.48	0.41	-	0.72	1.32	0.44	0.48	-	0.82	1.19
Bryophytes	0.39	0.33	0.34	0.50	1.02	0.34	0.36	0.32	0.52	0.97
Lichens	0.46	0.45	-	0.66	1.17	0.41	0.43	-	0.65	1.12
Phytophagous	0.37	0.42	0.37	0.38	0.81	0.31	0.38	0.40	0.39	0.87
Moths	0.34	0.28	-	0.35	0.76	0.33	0.30	-	0.35	0.74
Saproxylic	0.30	0.28	0.23	0.48	1.16	0.25	0.27	0.28	0.52	1.10
Fungi	0.37	0.31	0.33	0.31	0.76	0.28	0.33	0.34	0.31	0.80
Necrophagous	0.35	0.33	0.32	0.43	0.71	0.33	0.34	0.32	0.42	0.77
Spiders	0.45	0.25	0.29	0.38	0.78	0.37	0.29	0.34	0.39	0.79
Carabids	0.47	0.36	-	0.43	0.72	0.41	0.41	-	0.43	0.73
Birds	0.27	0.29	0.23	0.31	0.73	0.22	0.28	0.26	0.30	0.70
Bats	0.36	0.38	-	0.46	1.22	0.41	0.40	-	0.49	1.18
Median of 12 taxa	0.37	0.33	0.32	0.43	0.81	0.33	0.35	0.32	0.43	0.86

Supplementary Table 9 Cross-validated performance (R^2) of the assemblage habitat models (boosted GAMs) in the mixed effects models using the ALS and radar data sets. The construction of the mixed effects models included the effect of region as a random factor. R^2 was calculated based only on fixed factors, to exclude the region effect (random factor), denoted as Fixed (F), and only on a random factor, denoted as Random (R). F+R denote the aggregated R^2 based on both the F and the R factors.

		ALS models					Radar models				
		NMDS1	NMDS2	NMDS3	Species richness	Phylogenetic diversity	NMDS1	NMDS2	NMDS3	Species richness	Phylogenetic diversity
Plants	F+R	0.78	0.56	-	0.53	0.51	0.77	0.45	-	0.42	0.51
	Fixed	0.20	0.27	-	0.01	0.00	0.30	0.17	-	−0.05	0.12
	Random	0.70	0.28	-	0.40	0.51	0.67	0.28	-	0.41	0.48
Bryophytes	F+R	0.72	0.55	0.33	0.30	0.51	0.75	0.55	0.37	0.31	0.50
	Fixed	0.14	0.02	−0.02	0.01	0.12	0.34	0.03	0.03	0.01	0.13
	Random	0.68	0.55	0.37	0.31	0.48	0.63	0.55	0.35	0.31	0.47
Lichens	F+R	0.82	0.45	-	0.35	0.21	0.80	0.51	-	0.33	0.21
	Fixed	0.24	0.14	-	−0.01	0.14	0.36	0.11	-	0.03	0.21
	Random	0.75	0.36	-	0.32	0.07	0.69	0.37	-	0.30	−0.04
Phytophagous	F+R	0.82	0.65	0.48	0.34	0.50	0.82	0.65	0.46	0.20	0.48
	Fixed	0.30	0.02	0.14	0.11	0.04	0.48	0.07	0.10	0.05	0.01
	Random	0.72	0.65	0.32	0.19	0.48	0.66	0.64	0.33	0.17	0.47
Moths	F+R	0.78	0.74	-	0.29	0.23	0.78	0.72	-	0.19	0.32
	Fixed	0.07	0.15	-	0.02	0.12	0.10	0.13	-	-0.01	0.09
	Random	0.77	0.70	-	0.28	0.19	0.76	0.69	-	0.19	0.28
Saproxylic	F+R	0.80	0.60	0.56	0.44	0.48	0.80	0.52	0.51	0.34	0.47
	Fixed	0.32	0.12	0.07	0.23	0.21	0.53	0.14	0.05	0.04	0.30
	Random	0.67	0.40	0.49	0.30	0.38	0.56	0.41	0.48	0.31	0.32
Fungi	F+R	0.76	0.52	0.22	0.40	0.41	0.79	0.54	0.12	0.41	0.33
	Fixed	0.42	0.12	0.09	0.37	0.40	0.76	0.09	0.06	0.36	0.33
	Random	0.51	0.46	0.07	0.08	0.01	0.20	0.46	0.07	0.07	−0.01
Necrophagous	F+R	0.61	0.43	0.26	0.22	0.36	0.61	0.44	0.22	0.22	0.30
	Fixed	0.09	0.00	0.03	−0.04	0.16	0.18	0.06	0.00	0.01	0.06
	Random	0.58	0.44	0.23	0.22	0.27	0.56	0.41	0.22	0.20	0.28

Spiders	F+R	0.86	0.72	0.43	0.63	0.08	0.85	0.65	0.40	0.61	0.05
	Fixed	−0.04	0.61	0.00	0.03	0.04	0.04	0.55	0.02	0.11	0.00
	Random	0.85	0.25	0.35	0.57	0.04	0.84	0.21	0.35	0.57	0.04
Carabids	F+R	0.75	0.67	-	0.69	0.31	0.73	0.62	-	0.66	0.29
	Fixed	−0.02	0.35	-	0.08	0.01	0.04	0.27	-	0.02	−0.02
	Random	0.74	0.48	-	0.67	0.30	0.73	0.48	-	0.67	0.30
Birds	F+R	0.71	0.28	0.41	0.32	0.11	0.75	0.28	0.24	0.33	0.13
	Fixed	0.27	0.12	0.34	0.19	−0.02	0.62	0.17	0.20	0.31	0.08
	Random	0.59	0.10	0.04	0.11	0.10	0.40	0.11	0.05	0.04	0.06
Bats	F+R	0.69	0.43	-	0.48	0.12	0.65	0.42	-	0.44	0.13
	Fixed	0.06	0.00	-	0.05	−0.02	0.10	−0.05	-	−0.05	0.04
	Random	0.67	0.43	-	0.43	0.13	0.65	0.43	-	0.43	0.11

Supplementary Table 10 Loss of R^2 in the assemblage habitat models by region. The R^2 of the mixed effects models was subtracted, leaving only the fixed factors to predict the response variables, to exclude the explained variance by region (a random factor) from the R^2 of the fixed effects models.

	ALS models					Radar models				
	NMDS1	NMDS2	NMDS3	Species richness	Phylogenetic diversity	NMDS1	NMDS2	NMDS3	Species richness	Phylogenetic diversity
Plants	0.29	0.19	-	0.26	0.24	0.29	0.07	-	0.11	0.25
Bryophytes	0.25	0.30	0.09	0.15	0.12	0.21	0.14	0.14	0.07	0.18
Lichens	0.32	0.08	-	0.04	0.01	0.29	0.17	-	0.04	0.00
Phytophagous	0.26	0.21	0.14	0.07	0.23	0.21	0.28	0.06	0.08	0.14
Moths	0.31	0.26	-	0.03	0.11	0.33	0.21	-	0.01	0.08
Saproxylic	0.25	0.16	0.41	0.04	0.11	0.18	0.17	0.19	0.10	0.09
Fungi	0.20	0.23	0.02	0.02	0.00	0.02	0.17	−0.01	0.00	0.00
Necrophagous	0.26	0.24	0.03	0.08	0.10	0.25	0.14	0.05	0.08	0.08
Spiders	0.38	0.07	0.37	0.35	0.01	0.52	0.03	0.13	0.23	0.03
Carabids	0.27	0.13	-	0.30	0.05	0.40	0.09	-	0.36	0.06
Birds	0.28	0.05	0.03	0.05	0.04	0.08	0.02	0.00	0.00	0.01
Bats	0.48	0.13	-	0.11	0.03	0.30	0.10	-	0.12	0.04
Median of 48 responses					0.14					0.10

Supplementary Table 11 Permits received for fieldwork

Offices granting permits	Permit number
Regierungspräsidium Tübingen	55-8/8848.02-07;<br>55-3/8852.15
Thüringer Landesverwaltungsamt	13.4 64233/11-07SDH;<br>13.4 64233/08-08SDH
Landesumweltamt Brandenburg	R07/SOB-0907;<br>LFU-N1- 47 43 /128+5#69 122/2018
UNB Eisenach	63.2-15.02.00.17-38-2018
Umweltamt Eichsfelde	001-04-18/6-85/uni-München/BiodivExploratorien
Umweltamt Kyffhäuserkreis	III.3.3-364.53.1/2018-06-01_BiodivExplo_Ergänzung_Arthropoden;<br>III.3.3-364.53.1/2018-08-01_Biodiv Expl_Hummeln
Landkreis Nordhausen	60.1.55.400.622/0239-18
Landratsamt Unstrut-Hainich-Kreis	10491-18-301
Regierung von Oberbayern	55.1-8646-32-2013
Regierung von Niederbayern	55.1-8642.10-N 25
Regierung von Oberfranken	55.1-8622
Regierung von Unterfranken	RUF-55. 1.2 -8622.147-2-14-3

Supplementary Notes

Supplementary Note 1 Sources of species data and detailed descriptions of the sampling methods

S1.1 Data sources

The biodiversity data were based on the following data sets and publications:

Exploratories

Jung, Kirsten; Marco Tschapka (2018): Bat activity in all Exploratories, summer 2008, using acoustic monitoring . v1.1.4. Biodiversity Exploratories Information System. Dataset. https://www.bexis.uni-jena.de/PublicData/PublicData.aspx?DatasetId=19848

Tschapka, Marco; Swen Renner; Kirsten Jung (2018): Bird survey data 2008. v3.1.4. Biodiversity Exploratories Information System. Dataset. https://www.bexis.uni-jena.de/PublicData/PublicData.aspx?DatasetId=21446

Goßner, Martin; Markus Lange; Manfred Türke; Esther Pasalic; Wolfgang Weisser (2016): Window and ground traps on forest EPs in 2008 subset Coleoptera. v1.1.3. Biodiversity Exploratories Information System. Dataset. https://www.bexis.uni-jena.de/PublicData/PublicData.aspx?DatasetId=16866

Goßner, Martin; Manfred Türke; Markus Lange; Esther Pasalic; Wolfgang Weisser (2016): Window and ground traps on forest EPs in 2008 subset Araneae. v1.1.3. Biodiversity Exploratories Information System. Dataset. https://www.bexis.uni-jena.de/PublicData/PublicData.aspx?DatasetId=16868

Fischer, Markus (2017): Deadwood inhabiting fungi presence absence (2010, all forest EPs). v1.2.2. Biodiversity Exploratories Information System. Dataset. https://www.bexis.uni-jena.de/PublicData/PublicData.aspx?DatasetId=18547

Müller, Jörg; Steffen Boch; Markus Fischer (2016): Bryophyte diversity in forests. v1.6.8. Biodiversity Exploratories Information System. Dataset. https://www.bexis.uni-jena.de/PublicData/PublicData.aspx?DatasetId=4141

Boch, Steffen; Daniel Prati; Markus Fischer (2016): Lichen diversity in forests. v1.11.14. Biodiversity Exploratories Information System. Dataset. https://www.bexis.uni-jena.de/PublicData/PublicData.aspx?DatasetId=4460

Schäfer, Deborah; Steffen Boch; Markus Fischer (2017): Vegetation Records for Forest EPs, 2009 - 2016. v1.4.5. Biodiversity Exploratories Information System. Dataset. https://www.bexis.uni-jena.de/PublicData/PublicData.aspx?DatasetId=20366

Steigerwald-Project

Doerfler, I., Gossner, M.M., Müller, J., Seibold, S. & Weisser, W.W. (2018). Deadwood enrichment combining integrative and segregative conservation elements enhances biodiversity of multiple taxa in managed forests. *Biol. Conserv.*, 228, 70–78.

and further, unpublished data provided by Jörg Müller (joerg.mueller@npv-bw.bayern.de)

BIOKLIM-Project

Bässler, C., Müller, J. & Dziock, F. (2010). Detection of Climate-Sensitive Zones and Identification of Climate Change Indicators: A Case Study from the Bavarian Forest National Park. *Folia Geobot.*, 45, 163–182.

Bässler, C., Müller, J., Dziock, F. & Brandl, R. (2010). Effects of resource availability and climate on the diversity of wood-decaying fungi. *J. Ecol.*, 98, 822–832.

Moning, C., Werth, S., Dziock, F., Bässler, C., Bradtka, J., Hothorn, T., *et al.* (2009). Lichen diversity in temperate montane forests is influenced by forest structure more than climate. *For. Ecol. Manag.*, 258, 745–751.

Müller, J. & Brandl, R. (2009). Assessing biodiversity by remote sensing in mountainous terrain: the potential of LiDAR to predict forest beetle assemblages. *J. Appl. Ecol.*, 46, 897–905.

Müller, J., Mehr, M., Bässler, C., Fenton, M.B., Hothorn, T., Pretzsch, H., *et al.* (2012). Aggregative response in bats: prey abundance versus habitat. *Oecologia*, 169, 673–684.

Müller, J., Moning, C., Bässler, C., Heurich, M. & Brandl, R. (2009). Using airborne laser scanning to model potential abundance and assemblages of forest passerines. *Basic Appl. Ecol.*, 10, 671–681.

Raabe, S., Müller, J., Manthey, M., Dürhammer, O., Teuber, U., Göttlein, A., *et al.* (2010). Drivers of bryophyte diversity allow implications for forest management with a focus on climate change. *For. Ecol. Manag.*, 260, 1956–1964.

and further, unpublished data provided by Jörg Müller (joerg.mueller@npv-bw.bayern.de)

S1.2 Detailed description of species data sampling

Description of the sampling protocols of the Exploratories (Schwäbische Alb: ALB, Hainich: HAI, Schorfheide-Chorin: SCH), the Steigerwald Project (STE) and the BioKlim-Project of the Bavarian Forest NP (BAY).

The methods, grain size and time frame for species sampling, although standardised within each project, differ between projects, albeit to varying extents. To obtained comparable estimates of diversity, the data had to be similarly cropped while in each case retaining as much information as possible. If data on certain taxonomic groups were collected for several years, as was the case in Biodiversity Exploratories, for each region only the data of the year closest to the ALS flights were chosen. If, however, the sampling campaigns covered different periods within that year, our selection was not restricted to those months equally covered by all projects, as this might have excluded data obtained during the peak of species occurrence. An exception to this approach was moths (see below).

S1.2.1) Bats

Bats were recorded as part of the Exploratories study from 2008 to 2010 using a Pettersson D 1000× ultrasound detector (Pettersson Electronic AG, Uppsala, Sweden), during a combination of point stops and transect walks. The 100 m × 100 m plots were walked from corner to corner in a straight line. The survey time per edge was 6 min as was the time spent at each corner, resulting in a total survey time of 48 min per plot. Detector walks were conducted twice per summer and the data were analysed to the species level or sonotype using the software Avisoft SAS Lab Pro, versions 5.0.24 and onward (Raimund Specht, Avisoft Bioacoustics, Berlin Germany). Counts were defined as a minimum of two consecutive echolocation calls. Successive passes within 1 min were distinguished if the time interval between calls was larger than three times the regular pulse interval of the respective species (for details, see Jung, et al. [4]).

In the plots of the BioKlim and Steigerwald projects, autonomous bat call recorders (Batcorder 2.0; ecoObs GmbH, www.ecoobs.com) were placed as close to the middle of each plot as possible, at a height of ~2 m (STE) or ~2.7 m (BAY). The batcorder should cover a radius of ~20 m, but detectability differs between species. Bat sonotype and species were processed using the software bcAdmin1.11 (www.ecoobs.com), and species were identified using the software

bcDiscriminator1.14 (www.ecoobs.com). Counts were defined as the number of 1-min intervals per night in which a species was recorded. Bats were recorded in three rounds, from April to August 2017, in the Steigerwald plots and in seven rounds, from May to August 2009, in the BioKlim plots (for detailed information for BAY, see Müller, et al. [5]).

Not all bat species can be unambiguously identified to the species level based on their echolocation calls and thus are combined into sonotypes. The combination into sonotypes differs between regions. In the analysis, only species that could be identified to the species level were included. An exception was the sonotypes "Myotis brandtii_mystacinus" and "Plecotus", which could not be tracked to a single species by either of the two above-described softwares. In this study, sonotype was used as a species surrogate. Note, that although different bat detection systems were used, regional differences were much larger than the differences between the projects.

S1.2.2) Birds

In the Exploratories study, were monitored five times during the 2008–2010 the breeding seasons, between March and June. All bird hearings or sightings were recorded within 5 min and within a 50-m radius from plot midpoint (for detailed information, see Wells, et al. [6]).

In the Steigerwald forest and BioKlim Project, the procedures were similar but the recording time was 7 min in 2014 (STE) and 10 min in 2009 (BAY) and the distance was 1 ha (for detailed information for BAY, see Müller, et al. [7]).

In the regions used in the external validations (Bavarian Forest, Berchtesgaden, Brandenburg), similar point-counts sampling within a fixed radius of 50 m was conducted five times during the breeding season.

In the UNESCO-Biosphere reserve Rhön, another of the external validation regions, breeding birds were recorded by territory mapping following the methods of Fischer, et al. [8]. In short, all bird hearings and sightings were mapped, and territorial behaviour was recorded within several forested areas up to 25 ha in size. Sample areas were monitored seven times within the 2016–2018 breeding seasons (March–June), during which time data on territory status and breeding behaviour were also collected.

S1.2.3) Arthropods

Ground-dwelling arthropods were sampled using pitfall traps. In the Exploratories, these consisted of funnels (diameter: 15 cm) placed at three random corners of the plots from May to October in 2008 (pitfall traps had in some cases already been installed in April, but not in all plots; the records from April were excluded from the analysis). Two of the three funnels (denoted "Priority 1" and "Priority 2") were then randomly chosen for species determination (note that the designation of a trap as "Priority 1" or "Priority 2" could differ between months). In the Steigerwald forest and BioKlim project, only one trap, consisting of a plastic cup (diameter: ~ 8 cm), was established per plot and was placed near the midpoint of the plot. In the Steigerwald, the traps were operated from March to October 2016, and in the Bavarian Forest National Park from April to October 2007.

In addition to each pitfall trap, a flight interception trap, placed, at a height of ~1.5 m, was installed nearby. Like the procedure for the pitfall traps, only two of three traps were chosen for the monthly determinations and the Exploratories records from April were excluded. The flight interception traps of the projects were of the same size and type. However, those of the Exploratories had two sampling units, one at the bottom and one at the top of each trap, whereas in the Steigerwald and Bavarian Forest National Park each trap had one sampling unit, located at the bottom.

In both trap systems and across all projects, the trapping liquid consisted of copper sulphate, with a drop of detergent added to reduce the surface tension. The sampling vials were replaced once a month.

Moths were collected using the same trapping technique in all projects. Super-actinic UV-lights (12 V and 15 Watts) linked to a twilight-sensor and powered by a 12-V, 15-AH battery served as light traps. The traps were installed for two nights per plot between the end of May and mid-August (the phenological peak of moth occurrence). The catch was collected the next day and subsequently frozen before determination to the species level (carried out by Hermann Hacker).

For our analysis, only data from the "Priority 1" sample and ground window traps from the Exploratories were selected, such that the sampling sizes were the same as in the Steigerwald and Bavarian Forest National Park projects. If a "Priority 1" sample was not available for a given month, the "Priority 2" sample was used instead (for more information, see for example Lange,

et al. [9], Schall, et al. [10] [HAI, SCH, ALB], Doerfler, et al. [11], Roth, et al. [12] [both for STE] and Müller and Brandl [13] [BAY]).[13]

In the UNESCO-Biosphere Rhön, which served as an external validation region, beetles were obtained using flight interception traps and by hand collection. Two flight interception traps with sampling units at their bottom were installed at each study site at a height of ~1.5 m. The traps were operated from the end of April to October 2018. During this sampling period, beetles were also hand collected three times at each site, with special focus on deadwood structures, bark cracks etc. All samples were stored in ethanol until their determination. In DBU Natural Heritage areas, a flight interception trap was installed in the crown of a suitable tree, if possible close to deadwood and with optimal light exposure, in the centre of each forest stand. Samples from the trap were collected five times per year from May 2015 to October 2016.

In other external validation regions (Bavarian Forest, NPBBerchtesgaden), saproxylic beetles were collected using traps of the same type (flight interception traps and pitfall traps) as described for the training data regions.

Spider names were those listed in *wiki.arages (https://wiki.arages.de/, 30.09.2018)*, and beetle names were based on Entomofauna Germanica. Because Coleoptera is a hyper-diverse group that carries out several ecological functions, four subgroups were selected for this study: all beetles known to be saproxylic (based on Seibold, et al. [14]), species belonging to the family Carabidae, species known to be necrophagous and those known to be phytophagous (based on Koch 1992-1989). For moths, families belonging to the macrolepidoptera as well as to the Cossidae, Hepialidae and Limacodidae were chosen.

S1.2.4) Fungi

In the Exploratories, eleven deadwood objects located within the 1-ha plot were examined in 2010. Nine were randomly chosen and the other two were the largest objects on the plot. In the Steigerwald, a smaller, circular area of 0.1 ha was examined. Here, all deadwood objects and the soil were examined for 45 min in the spring, summer and autumn of 2014. In the Bavarian Forest National Park, an area of 0.1 ha was examined for 2 h in a single survey conducted from August to October 2006, during which time a minimum of the 15 most common deadwood objects but

as many of the remaining objects as possible were searched (also see Bässler, et al. [15] and Roth, et al. [12]).

Because of the extreme differences in the fungal communities of the different projects (Supplementary Figure5), which were unlikely to have been due to regional differences alone but no doubt also reflected the degree of determinability of cryptic species, the data were cropped to a list of species equally determinable by a specialist (C.B.). Thus, for instance, crustean species were not included. Species names were harmonised based on the *index fungorum* (http://www.indexfungorum.org/names/names.asp, 07.11.2018). Note that because the number of objects searched for fungi differed between the study sites, object-wise abundance data could not be compared.

S1.2.5) Lichens

In the Exploratories plots, lichens on four different substrates (bark [noting the tree species], rocks, deadwood and soil) within a 20 m × 20 m quadrats were recorded during a single session between 2007 and 2008[16]. Lichens were recorded in one round on all trees and dead wood stems and logs (hereafter referred to as stems) within a 14 m × 14 m plot in 2017 in the Steigerwald forest and within an 8-m radius from August to November 2007 in the Bavarian Forest National Park, respectively. In the Bavarian Forest National Park, 1–10 stems (average: 5 stems per plot) were examined, depending on stem availability (for details, see Moning, et al. [17]). Because lichens which were observed on rocks and soil were only recorded on the Exploratories plots, they were excluded from our analysis.

Species names were harmonised based on http://www.indexfungorum.org/. Because the number of objects searched for lichens differed between the projects, object-wise abundance data could not be compared.

S1.2.6) Bryophytes

In the Exploratories, all bryophytes within the core area (20 m × 20 m) of the plot were recorded and their abundance per substrate (soil, deadwood, bark and rock) was estimated between 2007 and 2008. In the Steigerwald, bryophytes were mapped within an area of 14 m × 14 m in April

2014. In the Bavarian Forest National Park, all bryophytes within a 0.02-ha circular plot were recorded in the summer of 2007. The abundance of bryophytes growing on the soil was estimated as a percentage of the cover. Bryophytes growing on deadwood were counted object-wise (see Raabe, et al. [18]). Note that, due to the use of different techniques in the estimation of abundance, only presence-absence data were used in our study. Species names were harmonised based on plantlist.org.

S1.2.7) Vascular plants

In the Exploratories study, vegetation has been recorded twice per year (spring and summer), on 20 m × 20 m quadrats, since 2009. For the ALB, species records from 2010 were chosen, and for HAI and SCH those of 2009. The percentage cover of single vascular plant species was estimated separately for two tree layers (5–10 m and >10 m), the shrub layer (including all woody species older than one year; one layer: 0–5 m), and the herb layer (including phanerophyte seedlings). In the Steigerwald, the vegetation within a 200-m² square was mapped in April and June 2014. Here, the herb layer included all plants with a height < 1.5 m, and the shrub layer all plants with heights of 1.5–5 m. Because several experts were involved in the determinations, species cover was assessed according to the Braun-Blanquet scale, which is not prone to overestimation of high covers. In the Bavarian Forest National Park, the vegetation in the summer of 2006 was recorded on a circular 0.2-ha plot according to the Londo scale. Here, the herb layer was defined as that below 1 m. In contrast to the other projects, only one mapping round was conducted. The presence of vernal geophytes was assumed to be negligible due to the short growing season and the absence of rich soils[19].

From the spring and summer records of ALB, HAI, SCH and STE, the record with the highest percentage cover was chosen. Plant names were harmonised based on plantlist.org.

Supplementary Note 2 Description of the calculations of phylogenetic diversity

S2.1) Vascular plants

From the 351 plants that could be identified to the species level, 349 could be matched with the phylogeny of Durka and Michalski [20]. The remaining two species, *Taraxacum campylodes* and *Taraxacum hamatum*, were added as replacements for the *Taraxacum* species for which no representative occurred in our data.

S2.2) Bryophytes

A phylogeny for bryophytes was constructed using the multiple sequence alignment (MSA) of Rose, et al. [21], which includes 21 gene regions. However, because some species in our dataset were not covered by this MSA, additional ITS gene region (5.8S rRNA) sequences for 89 species were searched in GenBank[22]. These additional sequences were aligned with the existing ITS alignment using MAFFT[23]. Species that were still not included in the resulting MSA were added randomly within the genus clade after the phylogeny inference. The 21 gene regions of the MSA were concatenated and the phylogeny then computed using RAxML (Randomized Axelerated Maximum Likelihood;[24], on the Cyberinfrastructure for Phylogenetic Research (CIPRES) science gateway server[25]) and the GTRCAT model. After an additional 100 rapid bootstraps with branch lengths estimation (flag -k), the final maximum likelihood phylogeny and each of the 100 bootstrap trees were pruned to match the species within our dataset. Seven species could not be added to the MSA or the phylogeny, because either no sequence was available or the respective genus was not represented in the phylogeny. To retrieve ultrametric trees for our analyses, divergence times were computed using the function *chronos* (penalised likelihood) from the R package *ape*[26]. The phylogenetic uncertainty within our analyses was assessed using the 100 bootstrap trees.

S2.3) Arthropods and lichens

The phylogeny for all beetle groups was created by pruning the phylogeny of Chesters [27] to all recorded beetle species. All missing species were added using the *addTip* function of *megaptera* v1.1.6, which adds a tip to the monophyletic generic clade of the respective species.

For spiders, *megaptera* (available on https://github.com/heibl/megaptera) was used to estimate the phylogeny based on the data matrix of published DNA sequences assembled at the GenBank[22] and Barcode of Life Data Systems (BOLD) repositories. Four mitochondrial and three nuclear loci were aligned using the L-INS-I algorithm (MAFFT)[23]. After the removal of ambiguously aligned nucleotide positions, the sequences were concatenated into a single matrix.

For moths, a phylogeny of 1355 Lepidoptera reported from Germany was constructed using *megaptera*. COI sequences were downloaded from GenBank, BOLD, and non-public sequences were provided by Advanced Identification Methods (AIM), Munich. Orthologous sequences were selected using the Basic Local Alignment Search Tool (BLAST); >98% of the species were represented by sequences, and the remaining 25 species were grafted onto the final tree according to their taxonomic position. Alignment and tree search were conducted using a super-familial backbone tree based on Mitter, et al. [28]. Shimodaira–Hasegawa likelihood ratio tests were performed to assess branch support (SH-aLRT branch support) using RAxML (flag -f J).

For lichens, the phylogeny published in Bässler, et al. [29] was updated and all available nuclear and mitochondrial sequences from the nucleotide repository of GenBank[22] were downloaded using E-utilities[30]. A heuristic algorithm was used to filter out dubious sequence records originating from the misidentification of specimens or from errors during DNA sequencing and data handling, including errors during data upload to National Center for Biotechnology Information (NCBI) repositories. A literature-based guide tree was used to successively align the remaining sequences of each genetic marker with the L-INS-i algorithm (MAFFT[23]). Non-aligned blocks of sequences were those having a pairwise genetic distance ≥0.5. This threshold is arbitrary, but a series of trials always led to the same arrangement of homologous alignment blocks that would have been generated by manual correction. After the removal of ambiguously aligned nucleotide positions (Gblocks 0.91b[31]), the alignment blocks of all markers and genomes were concatenated into a single super-matrix.

Tree topology and branch lengths for spiders, moths, and lichens were modelled in a maximum-likelihood framework (RAxML v8.4.2)[32] as implemented on XSEDE (https://www.xsede.org/) and accessed through the Cyberinfrastructure for Phylogenetic Research (CIPRES) science gateway[25]. Sequence evolution was modelled using the GTRCAT approximation for each marker gene separately on a common topology[32]. The tree topology confidence level was assessed using 1,000 non-parametric bootstrap replicates[33]. Branch lengths were converted from substitution per

site to a relative time scale, hence making the tree ultrametric, using the function *chronos* in the R package *ape*, with a penalised likelihood approach using a relaxed clock model of rate evolution and the smoothing parameter lambda = 1.0[26,34]. Because the phylogenetic information of the seven loci could not be expected to resolve deep nodes in the phylogeny, a topological constraint was imposed on the phylogeny based on recent phylogenomic studies[35]. For species without any available genetic information, congeners were used as proxies or, if this was not possible, these species were added randomly to the final topology of the respective genus, which had little effect on the analyses.

S2.4) Birds, bats, and fungi

Phylogenetic trees for birds were compiled following the phylogeny subset methodology from www.birdtree.org and used to mine 4,000 bootstrap trees, based on the backbone provided by Hackett, et al. [36]. Those bootstrap replicates were then condensed into a fully dated consensus tree using TreeAnnotator 1.8.2 (available on http://beast.community/treeannotator).

The phylogenetic trees compiled by Bässler, et al. [37] for fungi and by Riedinger, et al. [38] for bats were used in our study. If the published phylogenetic trees did not include all studied species, we employed the function *addTips* in *megaptera* v1.1.6 to add the missing tips using information from NCBI's taxonomy database.

Supplementary Table 12 References for the basic topologies of the phylogenetic trees of 12 functional groups

	References
Plants	Durka and Michalski [20]
Bryophytes	Rose, et al. [21]
Lichens	Bässler, et al. [29]
Phytophagous	Chesters [27]
Moths	Mitter, et al. [28]
Saproxylic	Chesters [27]
Fungi	Bässler, et al. [37]
Necrophagous	Chesters [27]
Aranea	-
Carabids	Chesters [27]
Birds	Hackett, et al. [36]
Bats	Riedinger, et al. [38]

Supplementary Note 3 Sentinel-1 data pre-processing and pixel-based summary statistics

S3.1 Download of the GRDH product from the ESA Sentinel Data Hub (DHuS)

All Sentinel data are available for free from the official ESA data portal (https:/scihub.copernicus.eu). User registration is required, but free. DHuS offers a user interface for interactive data discovery, or, alternatively an API for automated query and retrieval.

This manuscript was based on the Sentinel-1 GRDH product derived from inferometric wide swath acquisition mode (IW), which is a high-level product requiring only little further processing steps. It could be derived manually from lower level “Single Look Complex” data (SLC), however, since only backscatter coefficients were utilized in this manuscript, this was not necessary.

The GRDH product is provided in image coordinates (slant-range coordinates) as per original viewing geometry, including geometric and radiometric distortions and noise. The data are provided as uncalibrated digital numbers (DN).

S3.2 Process Sentinel-1 GRDH data to gamma_0

1. Required software

Processing of Sentinel-1 SAR data as utilized in this manuscript can be replicated by applying the provided batch processing configuration file for the SNAP toolbox software. The SNAP software is distributed officially by the European Space Agency (ESA); it is open-source and available free of charge, including detailed installation instructions, at https://step.esa.int/main/download/snap-download/.

Supported operating systems are Windows, Mac OS X and Unix (64bit).

2. The processing steps

All processing steps can be reproduced by means of the Graph Processing Tools (GPT), i.e. the command line interface included in the SNAP toolbox by applying the provided processing chain batch file (Sentinel1_GRDH_to_gamma0.xml).

The batch file contains placeholders for input and output files, which will be automatically replaced when using the corresponding command line flags as shown below:

Supplementary Table 13 Example for running the pre-processing from a batch command line.

```
## Configuration
inputFile=S1A...GRDH…SAFE.zip   # Downloaded GRDH file
outputFile=S1_gamma0.tif        # Output file

## Execute batch file with GPT
gpt -c 5G   \            # alotted memory for this process
    -q 4  \              # alotted CPUs for this process
    -x Sentinel1_GRDH_to_gamma0.xml \  # batch file containing processing chain
    -PinFile=$inputFile  \   # specify input file (downloaded GRDH dataset)
    -PoutFile=$outputFile \   # specify output file
```

The processing workflow as available in the xml batch file is as follows:

1) Apply precise orbit

This downloads the precise orbit state vectors at the time of data acquisition, which are needed for exact location of the satellite and hence the acquired SAR data. Precise orbit data are available approximately two weeks after data take.

This module only adds metadata and does not change the input.

2) Remove border noise

Sentinel-1 GRDH data exhibit strong artefacts at the swath borders. This module attempts to remove these artefacts within a search distance of 700 pixels from each margin.

The output of this module is in DN, without border noise.

3) Remove thermal noise

This module removes thermal detector noise.

The output of this module is in DN, with thermal noise removed.

4) Radiometric calibration

Radiometric calibration is needed to convert the SAR data from digital numbers (DNs), to calibrated backscatter intensities, which are comparable across acquisitions and sensors. Radiometric calibration output is in beta_0 (radar brightness coefficient), which is the required input data-level for radiometric terrain flattening.

5) Radiometric terrain flattening

Differences in terrain introduce not only differences in geolocation of SAR data, but also of the backscatter intensity. The aim of radiometric terrain flattening is to normalize the signal

intensity across differences in underlying terrain. To this end, it uses a digital elevation model (DEM), which in this study was the SRTM 1 sec DEM.

The output of this module is gamma_0 in slant-range coordinates.

6) Range-doppler terrain correction

The Sentinel GRDH product is not provided in geographic coordinates, but in image coordinates directly resulting from the SAR acquisition mode (slant-range coordinates). In order to exactly geolocate each pixel, and correct for geometric differences due to the local terrain, on needs to apply this geometric terrain correction or orthorectification. As with radiometric terrain flattening, this module relies also on a DEM, in combination with the precise orbit determination applied in step 1.

The output of this module is gamma_0 in geographic coordinates with 10 m pixel spacing.

Another step often useful for SAR data processing is spatial speckle filtering. However, due to the approach taken in this manuscript, that is, calculating median backscatter over multiple acquisitions, smoothing of speckle effects takes place implicitly in the time, instead of in space, thus not causing any further loss of geometric detail.

Processing time: 14 mins on a scene

Processing systems:

We tested the example on the following platform:

Windows edition: Windows 8.1 Enterprise

Processor: Intel(R) Core(TM) i7-6700 CPU @ 3.40GHz

RAM: 64.0 GB

System type: 64-bit Operating System, x64-based processor

S3.3 Derivation of pixel- and neighborhood-based summary statistics

This section builds on temporal stacks of Sentinel-1 gamma_0 data processed as described above. Like the rest of the manuscript it was performed using R.

1. Required software

Download and install R in following website depending on your operating system (Windows, Mac OS X, Linux) : https://cran.r-project.org/.

Minimum required packages which need to be installed in addition are: rgeos, raster, rgdal, glcm

2. Demo workflow

Define your input directory containing example input files.

The list of example input files is as follows:

1) Two temporal stacks of VH and VV gamma_0 data:
   ALB_gamma0_VH.tif and ALB_gamma0_VV.tif
2) Sentinel-1 data acquisition dates: ALB_acquisition_dates.csv
3) Shapefile of center of example plots: ALB_example_plots.*
4) Calculated mean values of all our 1-ha plots: S1_mean.csv

Source the R script. << Sentinel1_pixel_ neighborhood _statistics.r >>

Processing time: 1 mins

Code availability

The batch processing configuration file for the SNAP toolbox software and the R script for pixel- and summary statistics are publicly available at https://github.com/So-YeonBae/Sentinel1-Biodiversity and Supplementary Software.

Supplementary References

1 Dostálová, A., Wagner, W., Milenković, M. & Hollaus, M. Annual seasonality in Sentinel-1 signal for forest mapping and forest type classification. *International Journal of Remote Sensing* **39**, 7738-7760 (2018).

2 Huang, X., Ziniti, B., Torbick, N. & Ducey, M. J. R. S. Assessment of Forest above Ground Biomass Estimation Using Multi-Temporal C-band Sentinel-1 and Polarimetric L-band PALSAR-2 Data. **10**, 1424 (2018).

3 Chao, A. & Jost, L. Coverage-based rarefaction and extrapolation: standardizing samples by completeness rather than size. *Ecology* **93**, 2533-2547 (2012).

4 Jung, K., Kaiser, S., Böhm, S., Nieschulze, J. & Kalko, E. K. V. Moving in three dimensions: Effects of structural complexity on occurrence and activity of insectivorous bats in managed forest stands. *Journal of Applied Ecology* **49**, 523-531 (2012).

5 Müller, J. *et al.* Aggregative response in bats: prey abundance versus habitat. *Oecologia* **169**, 673-684 (2012).

6 Wells, K., Böhm, S. M., Boch, S., Fischer, M. & Kalko, E. K. V. Local and landscape-scale forest attributes differ in their impact on bird assemblages across years in forest production landscapes. *Basic and Applied Ecology* **12**, 97-106 (2011).

7 Müller, J., Moning, C., Bässler, C., Heurich, M. & Brandl, R. Using airborne laser scanning to model potential abundance and assemblages of forest passerines. *Basic and Applied Ecology* **10**, 671-681 (2009).

8 Fischer, S., Flade, M. & Schwarz, J. in *Methodenstandards zur Erfassung der Brutvögel Deutschlands* (ed Peter Südbeck) (Dachverband Deutscher Avifaunisten, 2005).

9 Lange, M. *et al.* Effects of forest management on ground-dwelling beetles (Coleoptera; Carabidae, Staphylinidae) in Central Europe are mainly mediated by changes in forest structure. *Forest Ecology and Management* **329**, 166-176 (2014).

10 Schall, P. *et al.* The impact of even-aged and uneven-aged forest management on regional biodiversity of multiple taxa in European beech forests. *Journal of Applied Ecology* **55**, 267-278 (2018).

11 Doerfler, I., Müller, J., Gossner, M. M., Hofner, B. & Weisser, W. W. Success of a deadwood enrichment strategy in production forests depends on stand type and management intensity. *Forest Ecology and Management* **400**, 607-620 (2017).

12 Roth, N. *et al.* Decadal effects of landscape-wide enrichment of dead wood on saproxylic organisms in beech forests of different historic management intensity. *Diversity and Distributions* **25**, 430-441 (2019).

13 Müller, J. & Brandl, R. Assessing biodiversity by remote sensing in mountainous terrain: the potential of LiDAR to predict forest beetle assemblages. *Journal of Applied Ecology* **46**, 897-905 (2009).

14 Seibold, S. *et al.* Association of extinction risk of saproxylic beetles with ecological degradation of forests in Europe. *Conservation Biology* **29**, 382-390 (2015).

15 Bässler, C., Müller, J., Dziock, F. & Brandl, R. Effects of resource availability and climate on the diversity of wood-decaying fungi. *Journal of Ecology* **98**, 822-832 (2010).

16 Boch, S., Prati, D., Hessenmöller, D., Schulze, E.-D. & Fischer, M. Richness of Lichen Species, Especially of Threatened Ones, Is Promoted by Management Methods Furthering Stand Continuity. *PLOS ONE* **8**, e55461 (2013).

17 Moning, C. *et al.* Lichen diversity in temperate montane forests is influenced by forest structure more than climate. *Forest Ecology and Management* **258**, 745-751 (2009).

18 Raabe, S. *et al.* Drivers of bryophyte diversity allow implications for forest management with a focus on climate change. *Forest Ecology and Management* **260**, 1956-1964 (2010).

19 Bässler, C., Müller, J. & Dziock, F. Detection of Climate-Sensitive Zones and Identification of Climate Change Indicators: A Case Study from the Bavarian Forest National Park. *Folia Geobotanica* **45**, 163-182 (2010).
20 Durka, W. & Michalski, S. G. Daphne: a dated phylogeny of a large European flora for phylogenetically informed ecological analyses. *Ecology* **93**, 2297-2297 (2012).
21 Rose, J. P., Kriebel, R. & Sytsma, K. J. Shape analysis of moss (Bryophyta) sporophytes: Insights into land plant evolution. *American journal of botany* **103**, 652-662 (2016).
22 Benson, D. A. *et al.* GenBank. *Nucleic acids research* **41**, D36-D42 (2013).
23 Katoh, K. & Standley, D. M. MAFFT multiple sequence alignment software version 7: improvements in performance and usability. *Mol Biol Evol* **30**, 772-780 (2013).
24 Stamatakis, A. RAxML version 8: a tool for phylogenetic analysis and post-analysis of large phylogenies. *Bioinformatics (Oxford, England)* **30**, 1312-1313 (2014).
25 Miller, M. A., Pfeiffer, W. & Schwartz, T. in *2010 gateway computing environments workshop (GCE).* 1-8 (Ieee).
26 Paradis, E., Claude, J. & Strimmer, K. APE: analyses of phylogenetics and evolution in R language. *Bioinformatics (Oxford, England)* **20**, 289-290 (2004).
27 Chesters, D. Construction of a Species-Level Tree of Life for the Insects and Utility in Taxonomic Profiling. *Systematic Biology* **66**, 426-439 (2017).
28 Mitter, C., Davis, D. R. & Cummings, M. P. Phylogeny and evolution of Lepidoptera. *Annual review of entomology* **62**, 265-283 (2017).
29 Bässler, C. *et al.* Contrasting patterns of lichen functional diversity and species richness across an elevation gradient. *Ecography* **39**, 689-698 (2016).
30 Sayers, E. The E-utilities in-depth: parameters, syntax and more. *Entrez Programming Utilities Help [Internet]* (2009).
31 Castresana, J. Selection of conserved blocks from multiple alignments for their use in phylogenetic analysis. *Molecular biology and evolution* **17**, 540-552 (2000).
32 Stamatakis, A. RAxML version 8: a tool for phylogenetic analysis and post-analysis of large phylogenies. *Bioinformatics* **30**, 1312-1313 (2014).
33 Stamatakis, A., Hoover, P. & Rougemont, J. A rapid bootstrap algorithm for the RAxML web servers. *Systematic biology* **57**, 758-771 (2008).
34 Paradis, E. Molecular dating of phylogenies by likelihood methods: a comparison of models and a new information criterion. *Molecular phylogenetics and evolution* **67**, 436-444 (2013).
35 Sharma, P. P. *et al.* Phylogenomic interrogation of Arachnida reveals systemic conflicts in phylogenetic signal. *Molecular Biology and Evolution* **31**, 2963-2984 (2014).
36 Hackett, S. J. *et al.* A Phylogenomic Study of Birds Reveals Their Evolutionary History. *Science* **320**, 1763-1768 (2008).
37 Bässler, C., Ernst, R., Cadotte, M., Heibl, C. & Müller, J. Near-to-nature logging influences fungal community assembly processes in a temperate forest. *Journal of Applied Ecology* **51**, 939-948 (2014).
38 Riedinger, V., Müller, J., Stadler, J., Ulrich, W. & Brandl, R. Assemblages of bats are phylogenetically clustered on a regional scale. *Basic and Applied Ecology* **14**, 74-80 (2013).
