# Supplementary material for: Radar vision in the mapping of forest biodiversity from space
Source: Nat Commun. 2019 Oct 18;10:4757. doi: 10.1038/s41467-019-12737-x (PMC6802221; doi:10.1038/s41467-019-12737-x)
Supplement: Supplementary file 3 — Description of Additional Supplementary Files [file 41467_2019_12737_MOESM3_ESM.pdf]

Description of Additional Supplementary Files

File Name: Supplementary Software 1
Description: Batch processing configuration file for the SNAP toolbox software and the R script for pixel- and summary statistics are provided at Supplementary Software as a ZIP file.
