# Supplementary material for: Radar vision in the mapping of forest biodiversity from space
Source: Nat Commun. 2019 Oct 18;10:4757. doi: 10.1038/s41467-019-12737-x (PMC6802221; doi:10.1038/s41467-019-12737-x)
Supplement: Supplementary file 4 — Supplementary Software 1 [file 41467_2019_12737_MOESM4_ESM.zip › Sentinel1_ExampleData2/Manual_Sentinel-1 data pre-processing and pixel-based summary statistics.pdf]

Radar vision in the mapping of forest biodiversity from space

Bae et al.

Supplementary Note 3 Sentinel-1 data pre-processing and pixel-based summary statistics

S3.1 Download of the GRDH product from the ESA Sentinel Data Hub (DHuS)

All Sentinel data are available for free from the official ESA data portal (https:/scihub.copernicus.eu). User registration is required, but free. DHuS offers a user interface for interactive data discovery, or, alternatively an API for automated query and retrieval.

This manuscript was based on the Sentinel-1 GRDH product derived from inferometric wide swath acquisition mode (IW), which is a high-level product requiring only little further processing steps. It could be derived manually from lower level “Single Look Complex” data (SLC), however, since only backscatter coefficients were utilized in this manuscript, this was not necessary.

The GRDH product is provided in image coordinates (slant-range coordinates) as per original viewing geometry, including geometric and radiometric distortions and noise. The data are provided as uncalibrated digital numbers (DN).

S3.2 Process Sentinel-1 GRDH data to gamma_0

1. Required software

Processing of Sentinel-1 SAR data as utilized in this manuscript can be replicated by applying the provided batch processing configuration file for the SNAP toolbox software. The SNAP software is distributed officially by the European Space Agency (ESA); it is open-source and available free of charge, including detailed installation instructions, at https://step.esa.int/main/download/snap-download/.

Supported operating systems are Windows, Mac OS X and Unix (64bit).

2. The processing steps

All processing steps can be reproduced by means of the Graph Processing Tools (GPT), i.e. the command line interface included in the SNAP toolbox by applying the provided processing chain batch file (Sentinel1_GRDH_to_gamma0.xml).

The batch file contains placeholders for input and output files, which will be automatically replaced when using the corresponding command line flags as shown below:

Table S 1 Example for running the pre-processing from a batch command line.

```
## Configuration
inputFile=S1A...GRDH…SAFE.zip   # Downloaded GRDH file
outputFile=S1_gamma0.tif        # Output file

## Execute batch file with GPT
gpt -c 5G   \             # alotted memory for this process
    -q 4   \              # alotted CPUs for this process
    -x Sentinel1_GRDH_to_gamma0.xml \  # batch file containing processing chain
    -PinFile=$inputFile  \   # specify input file (downloaded GRDH dataset)
    -PoutFile=$outputFile \  # specify output file
```

The processing workflow as available in the xml batch file is as follows:

1) Apply precise orbit

This downloads the precise orbit state vectors at the time of data acquisition, which are needed for exact location of the satellite and hence the acquired SAR data. Precise orbit data are available approximately two weeks after data take.

This module only adds metadata and does not change the input.

2) Remove border noise

Sentinel-1 GRDH data exhibit strong artefacts at the swath borders. This module attempts to remove these artefacts within a search distance of 700 pixels from each margin.

The output of this module is in DN, without border noise.

3) Remove thermal noise

This module removes thermal detector noise.

The output of this module is in DN, with thermal noise removed.

4) Radiometric calibration

Radiometric calibration is needed to convert the SAR data from digital numbers (DNs), to calibrated backscatter intensities, which are comparable across acquisitions and sensors. Radiometric calibration output is in beta_0 (radar brightness coefficient), which is the required input data-level for radiometric terrain flattening.

5) Radiometric terrain flattening

Differences in terrain introduce not only differences in geolocation of SAR data, but also of the backscatter intensity. The aim of radiometric terrain flattening is to normalize the signal intensity across differences in underlying terrain. To this end, it uses a digital elevation model (DEM), which in this study was the SRTM 1 sec DEM.

The output of this module is gamma_0 in slant-range coordinates.

6) Range-doppler terrain correction

The Sentinel GRDH product is not provided in geographic coordinates, but in image coordinates directly resulting from the SAR acquisition mode (slant-range coordinates). In order to exactly geolocate each pixel, and correct for geometric differences due to the local terrain, on needs to apply this geometric terrain correction or orthorectification. As with radiometric terrain flattening, this module relies also on a DEM, in combination with the precise orbit determination applied in step 1.

The output of this module is gamma_0 in geographic coordinates with 10 m pixel spacing.

Another step often useful for SAR data processing is spatial speckle filtering. However, due to the approach taken in this manuscript, that is, calculating median backscatter over multiple acquisitions, smoothing of speckle effects takes place implicitly in the time, instead of in space, thus not causing any further loss of geometric detail.

Processing time: 14 mins (on one scene)

Processing systems:

We tested the example on the following platform:

Windows edition: Windows 8.1 Enterprise

Processor: Intel(R) Core(TM) i7-6700 CPU @ 3.40GHz

RAM: 64.0 GB

System type: 64-bit Operating System, x64-based processor

S3.3 Derivation of pixel- and neighborhood-based summary statistics

This section builds on temporal stacks of Sentinel-1 gamma_0 data processed as described above. Like the rest of the manuscript it was performed using R.

1. Required software

Download and install R in following website depending on your operating system (Windows, Mac OS X, Linux) : https://cran.r-project.org/.

Minimum required packages which need to be installed in addition are: rgeos, raster, rgdal, glcm

2. Demo workflow

Define your input directory containing example input files.

The list of example input files is as follows:

1) Two temporal stacks of VH and VV gamma_0 data:
ALB_gamma0_VH.tif and ALB_gamma0_VV.tif
2) Sentinel-1 data acquisition dates: ALB_acquisition_dates.csv
3) Shapefile of center of example plots: ALB_example_plots.*
4) Calculated mean values of all our 1-ha plots: S1_mean.csv

Source the R script. << Sentinel1_pixel_ neighborhood _statistics.r >>

Processing time: 1 mins

Code availability

The batch processing configuration file for the SNAP toolbox software and the R script for pixel- and summary statistics are publicly available at https://github.com/So-YeonBae/Sentinel1-Biodiversity and Supplementary Software.
