# Supplementary material for: Radar vision in the mapping of forest biodiversity from space
Source: Nat Commun. 2019 Oct 18;10:4757. doi: 10.1038/s41467-019-12737-x (PMC6802221; doi:10.1038/s41467-019-12737-x)
Supplement: Supplementary file 5 — Reporting Summary [file 41467_2019_12737_MOESM5_ESM.pdf]

nature research

Corresponding author(s): Soyeon Bae

Last updated by author(s): Aug 29, 2019

Reporting Summary

Nature Research wishes to improve the reproducibility of the work that we publish. This form provides structure for consistency and transparency in reporting. For further information on Nature Research policies, see Authors & Referees and the Editorial Policy Checklist.

Statistics

For all statistical analyses, confirm that the following items are present in the figure legend, table legend, main text, or Methods section.

n/a	Confirmed	
☐	☒	The exact sample size (*n*) for each experimental group/condition, given as a discrete number and unit of measurement
☐	☒	A statement on whether measurements were taken from distinct samples or whether the same sample was measured repeatedly
☐	☒	The statistical test(s) used AND whether they are one- or two-sided<br>*Only common tests should be described solely by name; describe more complex techniques in the Methods section.*
☐	☒	A description of all covariates tested
☐	☒	A description of any assumptions or corrections, such as tests of normality and adjustment for multiple comparisons
☐	☒	A full description of the statistical parameters including central tendency (e.g. means) or other basic estimates (e.g. regression coefficient) AND variation (e.g. standard deviation) or associated estimates of uncertainty (e.g. confidence intervals)
☐	☒	For null hypothesis testing, the test statistic (e.g. *F*, *t*, *r*) with confidence intervals, effect sizes, degrees of freedom and *P* value noted<br>*Give P values as exact values whenever suitable.*
☒	☐	For Bayesian analysis, information on the choice of priors and Markov chain Monte Carlo settings
☒	☐	For hierarchical and complex designs, identification of the appropriate level for tests and full reporting of outcomes
☐	☒	Estimates of effect sizes (e.g. Cohen's *d*, Pearson's *r*), indicating how they were calculated

Our web collection on statistics for biologists contains articles on many of the points above.

Software and code

Policy information about availability of computer code

Data collection	The Sentinel Application Platforms (SNAP) Sentinel-1 Toolbox software (http://step.esa.int) was used in the radar data processing.<br>Metrics-calculations of radar textural variables were performed in R, version 3.4.0, using the R package glcm.<br>LAStools (http://lastools.org) and the R package lidR were used in the processing and metrics-calculation of airborne laser scanning.<br>The software Avisoft SAS Lab Pro, versions 5.0.24 and onward (Raimund Specht, Avisoft Bioacoustics, Berlin Germany) , bcAdmin1.11 and bcDiscriminator1.14 (www.ecoobs.com) were used in processing and identifying bat species.
Data analysis	The R package goeveg, vegan and picante was used in calculating biodiversity variables, and the R package candisc in a canonical correlation analysis, the R package mboost and dismo in boosted generalised additive models.<br>The megaptera (https://github.com/heibl/megaptera), bold (https://github.com/heibl/bold), TreeAnnotator (http://beast.community/treeannotator), RAxML (Randomized Axelerated Maximum Likelihood) and the R package ape were used in phylogenetic analyses.

For manuscripts utilizing custom algorithms or software that are central to the research but not yet described in published literature, software must be made available to editors/reviewers. We strongly encourage code deposition in a community repository (e.g. GitHub). See the Nature Research guidelines for submitting code & software for further information.

Data

Policy information about availability of data

All manuscripts must include a data availability statement. This statement should provide the following information, where applicable:

- Accession codes, unique identifiers, or web links for publicly available datasets
- A list of figures that have associated raw data
- A description of any restrictions on data availability

The data that support the findings of this study are publicly available on the BExIS platform, dataset ID 25206 (https://www.bexis.uni-jena.de/PublicData/PublicDataSet.aspx? DatasetId=25206). The source data underlying Figures 1 and 4 and Supplementary Figures 2, 5, 18 and 19 are provided as a Source Data file.

Field-specific reporting

Please select the one below that is the best fit for your research. If you are not sure, read the appropriate sections before making your selection.

☐ Life sciences ☐ Behavioural & social sciences ☒ Ecological, evolutionary & environmental sciences

For a reference copy of the document with all sections, see nature.com/documents/nr-reporting-summary-flat.pdf

Ecological, evolutionary & environmental sciences study design

All studies must disclose on these points even when the disclosure is negative.

Study description	Using the radar data by the Sentinel-1 mission, we conducted the evaluation of Sentinel-1's potential in biodiversity mapping. Our study began with a comparison of the ecological application of radar (henceforth, "Sentinel-1" is referred to as "radar") metrics vs. the ALS metrics in providing a better understanding of habitat structure in forest ecosystems. A suite of ground-truth biodiversity measures covering a broad range of trophic levels and taxa (henceforth "functional groups") was then modelled using either ALS data or radar data. We quantified the predictive power of radar in modelling different aspects of biodiversity and compared the results to those obtained using ALS data. For this purpose, we made use of a distributed ground-based network of 463 biodiversity monitoring plots spanning five Central European temperate forest regions and capturing biodiversity data for 12 functional groups. Finally, to test their suitability for biodiversity mapping and monitoring, the radar models for two taxa were validated using independent external data collected from areas outside the five training areas.
Research sample	We sampled a broad range of trophic levels and taxa: plants, bryophytes, lichens, phytophagous beetles, moths, saproxylic beetles, fungi, spiders, carabids, necrophagous beetles, birds and bats.<br>Concerning the remote sensing data, we sampled data derived from ALS and Sentinel-1. ALS is selected, as it has already well-established metrics describing habitat structure in forest ecosystems.<br>The biodiversity data were compiled from three well-established long-term biodiversity monitoring projects (Biodiversity Exploratories, the BIOKLIM Project and the Steigerwald Project). The data sets and related publications on which the biodiversity data were based were described in Supplementary Method S1.1.
Sampling strategy	The study was conducted at up to 463 plots in five forest regions distributed from north to south in Germany and representative of forest habitat types in Central Europe.<br>By compiling the data from the three biodiversity projects, the sample sizes were statistically sufficient for our analysis. For the analysis of each functional group, plots for which observations of the corresponding group were available were selected; the number of investigated plots per group was 454 for plants, 298 for bryophytes, 290 for lichens, 362 for phytophagous beetles, 219 for moths, 361 for saproxylic beetles, 458 for fungi, 361 for spiders, 347 for carabids, 334 for necrophagous beetles, 456 for birds and 201 for bats.
Data collection	The detailed description of species data sampling was described in Supplementary Method S1.2.<br>Data recorders of species data differed by taxa and projects and were written in Supplementary Method S1.1.<br>Concerning radar data, C-band synthetic aperture radar (C-SAR) data acquired by the Sentinel-1 mission throughout Germany were used in this study. The C-SAR data were acquired in interferometric wide mode in two polarisations, VV (vertically-sent, vertically-received radar pulse) and VH (vertically-sent, horizontally-received radar pulse), during both ascending and descending orbits. The ground-range-detected high-resolution product (GRDH), with a pixel spacing of 10 m, was downloaded from the ESA Scientific Hub. Airborne laser scanning (ALS) data were acquired by mainly Riegl Q560 with flight heights between 350-700 m a.g.l. (see Supplementary Table S2).
Timing and spatial scale	The period of data collection and frequency differ by taxa. The methods, grain size and time frame for species sampling, although standardised within each project, differ between projects, albeit to varying extents. Detailed timing and spatial scale per taxa and projects were described in Supplementary Method S1.2.<br>Concerning the radar data, we obtained the year data of 2016, as the year of 2016 is the closest year to the species sampling period and the first year to cover a complete year across our five regions after the launch of Sentinel-1 mission. Airborne laser scanning (ALS) data were acquired during leaf-on periods between 2007 and 2018, depending on the region (see Supplementary Table S2).
Data exclusions	The methods, grain size and time frame for species sampling, although standardised within each project, differ between projects, albeit to varying extents. To obtained comparable estimates of diversity, the data had to be similarly cropped while in each case retaining as much information as possible. If data on certain taxonomic groups were collected for several years, as was the case in Biodiversity Exploratories, for each region only the data of the year closest to the ALS flights were chosen.<br>From the BIOKLIM Project, conducted in the Bavarian Forest National Park, 244 plots among the 293 sampling plots were selected; the 49 excluded plots were those in which the change in canopy cover between 2007 (year of ALS acquisition) and 2016 (year of radar acquisition) exceeded 20% due to disturbances such as bark beetle infestation and wind-throw.
Reproducibility	Not applicable.
Randomization	In biodiversity modelling, five-fold cross-validations were performed; for each one, the region served as the sub-group, achieved by separating the training and test datasets by region and then combining the respective datasets to obtain total training and total test datasets for the five forest regions. To make use of the full range of environmental spaces and species pools covering all the gradients of the five regions, training and test data were extracted from all five regions.
Blinding	Not applicable. We used the species data already sampled from the three biodiversity monitoring projects, so there cannot be bias by

Blinding	analysts. While processing the remote sensing data and conducting our statistical analysis, it is hard that the subjectivity can be involved.

Did the study involve field work? ☐ Yes ☒ No

Reporting for specific materials, systems and methods

We require information from authors about some types of materials, experimental systems and methods used in many studies. Here, indicate whether each material, system or method listed is relevant to your study. If you are not sure if a list item applies to your research, read the appropriate section before selecting a response.

Materials & experimental systems

n/a	Involved in the study
☒	☐ Antibodies
☒	☐ Eukaryotic cell lines
☒	☐ Palaeontology
☐	☒ Animals and other organisms
☒	☐ Human research participants
☒	☐ Clinical data

Methods

n/a	Involved in the study
☒	☐ ChIP-seq
☒	☐ Flow cytometry
☒	☐ MRI-based neuroimaging

Animals and other organisms

Policy information about studies involving animals; ARRIVE guidelines recommended for reporting animal research

Laboratory animals	The study did not involve laboratory animals.
Wild animals	Bats and birds were recorded using acoustic monitoring and, for the latter, sightings as well. Plants, bryophytes, fungi and lichens were mapped in the field. For quantitative and qualitative recordings of Arthropods, we used flight-interception traps and pitfall traps with sulphate solution as trapping and killing liquid. Moths were caught by of 12 V and 15 Watt super actinic UV lighttraps, with chloroform as killing medium.
Field-collected samples	The sampled arthropods were preserved in alcohol of frozen until they could be determined by specialists.
Ethics oversight	We complied with all relevant ethical regulations for animal research. All the records of species, except for arthropods, were conducted by sightings or sound-recording in the field. The methods used in this study to assess arthropod diversity were legally mandated by the field work permits listed in the acknowledgement section and Supplementary Table 11. Fieldwork permits were issued by the responsible state environmental offices of Baden-Württemberg, Thüringen, Brandenburg and Bayern (See Supplementary Table 11 for details of permits).

Note that full information on the approval of the study protocol must also be provided in the manuscript.
